# Morphological Characteristics and Comparative Transcriptome Analysis of Three Different Phenotypes of *Pristella maxillaris*

**DOI:** 10.3389/fgene.2019.00698

**Published:** 2019-08-02

**Authors:** Fangfang Bian, Xuefen Yang, Zhijie Ou, Junzhi Luo, Bozhen Tan, Mingrui Yuan, Tiansheng Chen, Ruibin Yang

**Affiliations:** ^1^Key Laboratory of Freshwater Animal Breeding, Ministry of Agriculture, College of Fisheries, Huazhong Agricultural University, Wuhan, China; ^2^Department of Fisheries, Guangdong Maoming Agriculture & Forestry Technical College, Maoming, China; ^3^Collaborative Innovation Center for Efficient and Health Production of Fisheries in Hunan Province, Changde, China

**Keywords:** Pristella maxillaris, RNA-seq, melanophores, iridophores, molecular mechanism

## Abstract

*Pristella maxillaris* is known as the X-ray fish based on its translucent body. However, the morphological characteristics and the molecular regulatory mechanisms of these translucent bodies are still unknown. In this study, the following three phenotypes, a black-and-gray body color or wild-type (WT), a silvery-white body color defined as mutant I (MU1), and a fully transparent body with a visible visceral mass named as mutant II (MU2), were investigated to analyze their chromatophores and molecular mechanisms. The variety and distribution of pigment cells in the three phenotypes of *P. maxillaris* significantly differed by histological assessment. Three types of chromatophores (melanophores, iridophores, and xanthophores) were observed in the WT, whereas MU1 fish were deficient in melanophores, and MU2 fish lacked melanophores and iridophores. Transcriptome sequencing of the skin and peritoneal tissues of *P. maxillaris* identified a total of 166,089 unigenes. After comparing intergroup gene expression levels, more than 3,000 unigenes with significantly differential expression levels were identified among three strains. Functional annotation and Gene Ontology (GO) and Kyoto Encyclopedia of Genes and Genomes (KEGG) pathway analyses of the differentially expressed genes (DEGs) identified a number of candidates melanophores and iridophores genes that influence body color. Some DEGs that were identified using transcriptome analysis were confirmed by quantitative real-time PCR. This study serves as a global survey of the morphological characteristics and molecular mechanism of different body colors observed in *P. maxillaris* and thus provides a valuable theoretical foundation for the molecular regulation of the transparent phenotype.

## Introduction

As one of the most diverse phenotypic traits under strong selection pressure in many organisms, coloration plays numerous adaptive functions such as predator deterrence, species recognition, and even protecting the organism from solar ultraviolet radiation damage (Lowe and Goodman-Lowe, 1996; Parichy, 2006; Roberts et al., 2009; Zhang et al., 2015). Skin coloration can be influenced by many factors, such as genetics, diet, and general health (Wang et al., 2014; Zhang et al., 2015). Nevertheless, genetics remains the major determining factor (Braasch et al., 2007). Most animals have different body colors that are mainly determined by diverse pigments synthesized by chromatophores or pigment cells. Chromatophores are cells that are specialized in the storage and/or synthesis of light-absorbing pigments or light-reflecting structures (Bagnara et al., 2007; Leclercq et al., 2010; Henning et al., 2013). Teleost fishes have more than six types of pigment cells (Michiels et al., 2008; Wucherer and Michiels, 2012; Goda et al., 2013; Wucherer and Michiels, 2014; Schartl et al., 2016). Zhang et al. (2015) found that the differences in two color patterns of the crimson snapper (*Lutjanus erythropterus*) primarily depended on the density and distribution of pigment cells; in black skin, melanophores are the major pigment cells, and in red skin, iridophores and xanthophores are the major pigment cells. In adult red crucian carp (*Carassius auratus*, red var.), body color undergoes a gray-to-red change, which is due to alterations in the number of skin melanophores (Zhang et al., 2017). In addition, several studies have reported that many fish species can change body transparency based on the differentiation and development of chromatophores (Nilsson Skold et al., 2010; Krauss et al., 2013; Franco-Belussi et al., 2016). Some model fish mutants, such as medaka and zebrafish, exhibit a transparent phenotype by regulating the expression levels of some genes (Parichy, 2006; White et al., 2008; Krauss et al., 2013; Kimura et al., 2017). The differentiation and development of pigment cells are strictly regulated by genes (Roberts et al., 2009; Dooley et al., 2013), whereas the genetics behind natural color morph variants in ﬁsh remains largely unknown.

RNA-Seq analysis is the most convenient method to investigate gene expression patterns in organisms. To date, several studies have revealed gene expression profiles that are responsible for different color patterns in freshwater fish. The striped pattern of the zebrafish (*Danio rerio*) is generated by self-organizing mechanisms that require interactions among three different types of pigment cells (Irion et al., 2016). In addition, transcriptome analyses of different colored varieties of the common carp (*Cyprinus carpio* var. *color*) (Wang et al., 2014), crimson snapper (Zhang et al., 2015), Midas cichlid (*Amphilophus citrinellus*) (Higdon et al., 2013), and red crucian carp (Zhu et al., 2018) were performed to understand the genetic basis of coloration. Signaling pathways, such as the Wnt/β-catenin (wingless-type MMTV integration site family), tyrosinase synthesis, MAPK (mitogen-activated protein kinase), and cAMP (cyclic adenosine monophosphate) pathways, have been shown as conserved pathways that are related to melanophore development in vertebrates (Jiang et al., 2014; Wang et al., 2014). Several studies have investigated the regulatory mechanism of melanophore development, and many pigment-related genes have been identified in mice and fish (Newton et al., 2005; Hoekstra et al., 2006; Fang et al., 2018). However, only a few studies have investigated the role of iridophores (Ng et al., 2009; Higdon et al., 2013) and xanthophores (Sefc et al., 2014) in body coloration, and their detailed molecular mechanism has been less investigated.

*Pristella maxillaris*, also known as the X-ray fish, is a warm water fish belonging to the family Characidae and the order Characiformes. It is a widely distributed and adaptable fish found in the Amazon and Orinoco basins, as well as in coastal rivers in the Guianas. Due to its translucent body color, *P. maxillaris* is a valuable, ornamental fish that has a huge market. Morphological diversification of *P. maxillaris* has produced many kinds of transparent body mutations leading to coloration ranging from a black-gray body color to entirely transparent. There are three typical phenotypes: wild-type (WT), which has a black-and-gray body color with black spots on the trailing edge and fin of the operculum; mutant I (MU1), which has a silvery-white body color; and mutant II (MU2), which is fully transparent and has clearly observed visceral tissues. To date, most studies on *P. maxillaris* have mainly focused on their growth and development (Yu et al., 2018). However, investigations on body transparency mutations and the molecular mechanism in *P. maxillaris* have not yet been conducted. To better understand how cells and genetic factors alter body transparency, we utilized stereomicroscopy to observe the differences in chromatophores in WT fish and two different mutants, namely, MU1 and MU2. RNA-Seq was conducted on samples from the three phenotypes to compare their gene expression profiles. In particular, the signaling pathways and candidate genes whose mutations are responsible for differences in body transparency were also examined and quantified. The purpose of this study was to provide a global survey of the morphological characteristics and molecular mechanism of the different body colors in *P. maxillaris*, as well as generate theoretical foundation for the molecular regulation of the transparent phenotype.

## Materials and Methods

### Ethics Statement

No specific permissions were required for the use of *P. maxillaris* collected for this study in China. All the experimental procedures involving fish were approved by the Institutional Animal Care and Use Committee of Huazhong Agricultural University.

### Samples for Microscopy and Transcriptome Analysis

Samples from fish exhibiting three *P. maxillaris* phenotypes (WT, MU1, and MU2) were collected from the Flower and Bird Market in Wuhan, Hubei, China. Prior to the experiments, the fish were kept in laboratory aquariums under 14:10 h light/dark conditions at temperatures of 24 ± 2°C for 2 weeks to acclimate them to the experimental conditions. The fish were anesthetized in well-aerated water containing 100 mg/L tricaine methanesulfonate (MS-222) before being immediately euthanized. Six adult individuals exhibiting each *P. maxillaris* phenotype (average length, 3.5 ± 0.3 cm) were selected. The fresh pieces of the operculum lining, peritoneum, and skin were surgically excised and temporarily mounted for subsequent light microscopic (OLYMPUS SZX16) observation. In addition, fish exhibiting the three phenotypes were anesthetized and fixed for 24 h in formalin, and areas of approximately 1 cm^2^ in size were cut from the skin and peritoneal tissues for paraffin sectioning. The types and distributions of pigment cells in fish exhibiting the three different phenotypes were observed under a microscope (Imager A2). Pigment cell types are easily identified due to their colors and shapes by microscopic and histological methods based on the literatures. Melanophores show black/gray color and stellated shape; xanthophores exhibit yellow, orange, and red colors; iridophores contain white, blue, and purple-red color (Kelsh, 2004; Darias et al., 2013; Zhang et al., 2015; Zhang et al., 2017).

Additional skin and peritoneal tissues from different phenotypes were collected to extract total RNA, and we pooled the skin and peritoneal tissues from multiple individuals of each phenotype of *P. maxillaris*. All fresh tissue samples were frozen immediately in liquid nitrogen and then stored at −80°C before RNA isolation.

### RNA Extraction

Total RNA was obtained from the mixed samples of skin and peritoneum from fish exhibiting the three different phenotypes of *P. maxillaris* using RNAiso Plus Reagent (TaKaRa, China) according to the manufacturer’s protocol. Total RNA was extracted with a Qubit^®^ RNA Assay Kit in a Qubit^®^ 2.0 Fluorometer (Life Technologies, CA, USA). The RNA Nano 6000 Assay Kit from the Agilent Bioanalyzer 2100 system (Agilent Technologies, CA, USA) and gel electrophoresis were used to assess the quantity and quality of the total RNA.

mRNA was purified from the total RNA using polyT oligo-attached magnetic beads (NEB, USA). Fragmentation was carried out using divalent cations under elevated temperature in NEB-Next First-Strand Synthesis Reaction Buffer (5×). First-strand cDNA was synthesized using random hexameric primers and M-MuLV reverse transcriptase (RNase H). Second-strand cDNA synthesis was subsequently performed using DNA polymerase I and RNase H. The remaining overhangs were converted into blunt ends *via* exonuclease/polymerase activities. After adenylation of the 3’ ends of the DNA fragments, NEB Next Adaptor with hairpin loop structure was ligated to prepare for hybridization. cDNA fragments 250 to 300 bp long were selected as templates. An Agilent 2100 Bioanalyzer (Agilent Technologies, Santa Clara, CA, USA) and an AMPure XP real-time PCR system (Beckman Coulter, Beverly, USA) were used to quantify and qualify the sample library.

### Sequencing, Assembly, and Annotation

Transcriptome sequencing was conducted on an Illumina HiSeq 2000 RNA-Seq platform (Illumina, San Diego, CA, USA). Clean reads were acquired after removing reads with adapters, reads with more than 5% unknown nucleotides, and reads with a percentage of low-quality bases (base quality ď 10) more than 20%. Trinity was used to conduct the *de novo* assembly of the transcriptome (Grabherr et al., 2011). Contigs, longer fragments without N, were obtained by combining overlapping reads. Then, different contigs were connected to obtain sequences that could not be extended on either end, which were defined as unigenes. The assembled sequences were compared against the NCBI non-redundant (Nr) protein database, Swiss-Prot, Kyoto Encyclopedia of Gene and Genomes (KEGG), and the Clusters of Orthologous Groups (COG) database using BLASTX with an E-value of 1 × 10^-5^. The directions of the contig sequences were based on the best alignment results. A combination of the BLAST, Blast2GO, KEGG, and GO databases was used for functional annotation. BLASTX alignment (E-value < 1 × 10^-5^) with the NT, NR, KEGG, Swiss-Prot, and COG databases was conducted to obtain the associated gene name and gene ontology (GO) term accession number, and GO analysis was performed with WEGO software (Ye et al., 2006).

### Differential Gene Expression Analysis

Differential expression analysis between each pair of samples was performed using the DEG-seq R package (Robinson et al., 2010). P values were adjusted using q values (Shannon et al., 2003). The threshold for significantly differential expression was set at a q value < 0.005 and |Log2(fold change)| > 1. Based on the hypergeometric distribution model, GO and KEGG ontology enrichment analyses were conducted on the differentially expressed genes (DEGs). GO enrichment analysis of the DEGs was implemented by the GO-seq R package-based Wallenius noncentral hypergeometric distribution (Young et al., 2010), which can adjust for gene length bias in DEGs. KEGG is a database resource used to understand high-level functions and utilities of biological systems such as the cell, organism, and ecosystem from information at the molecular level, especially large-scale molecular data sets generated by genome sequencing and other high-throughput experimental technologies (http://www.genome.jp/kegg/). We used KOBAS software to test the statistical enrichment of DEGs in KEGG pathways (Mao et al., 2005).

### Quantitative Real-Time PCR Validation

We selected some genes randomly to validate the transcriptome data by using qRT-PCR with *gapdh* as an internal control. First-strand cDNA was obtained from the total RNA using random primers and the MMLV reverse transcriptase (Promega, Madison, WI, USA). Primers (listed in **Table S1**) were designed using Beacon software. The qRT-PCR was performed with SYBR Green PCR Super Mix (Thermo Scientific, Wilmington, DE, USA) and the CFX96 real-time PCR detection system (Bio-Rad, Hercules, CA, USA). PCR was performed in a 10-µl reaction volume containing 0.5 µl of each primer (5 µM), 0.5 µl cDNA, 5 µl SYBR Green Super Mix, and 3.5 µl ddH_2_O. The PCR cycle was performed as follows: 95°C for 7 min, followed by 40 cycles of 95°C for 10 s, 55°C for 15 s, and 72°C for 15 s. Three technical replicates and three biological replicates of each sample were run along with the internal control gene. Differences in the expression levels of the WT, MU1 and MU2 fish were assessed after first normalizing expression levels to those of *gapdh*, followed by log transformation.

## Results

### Differences in Chromatophores Among Three Different Phenotypes of *P. maxillaris*

**Figure 1:**
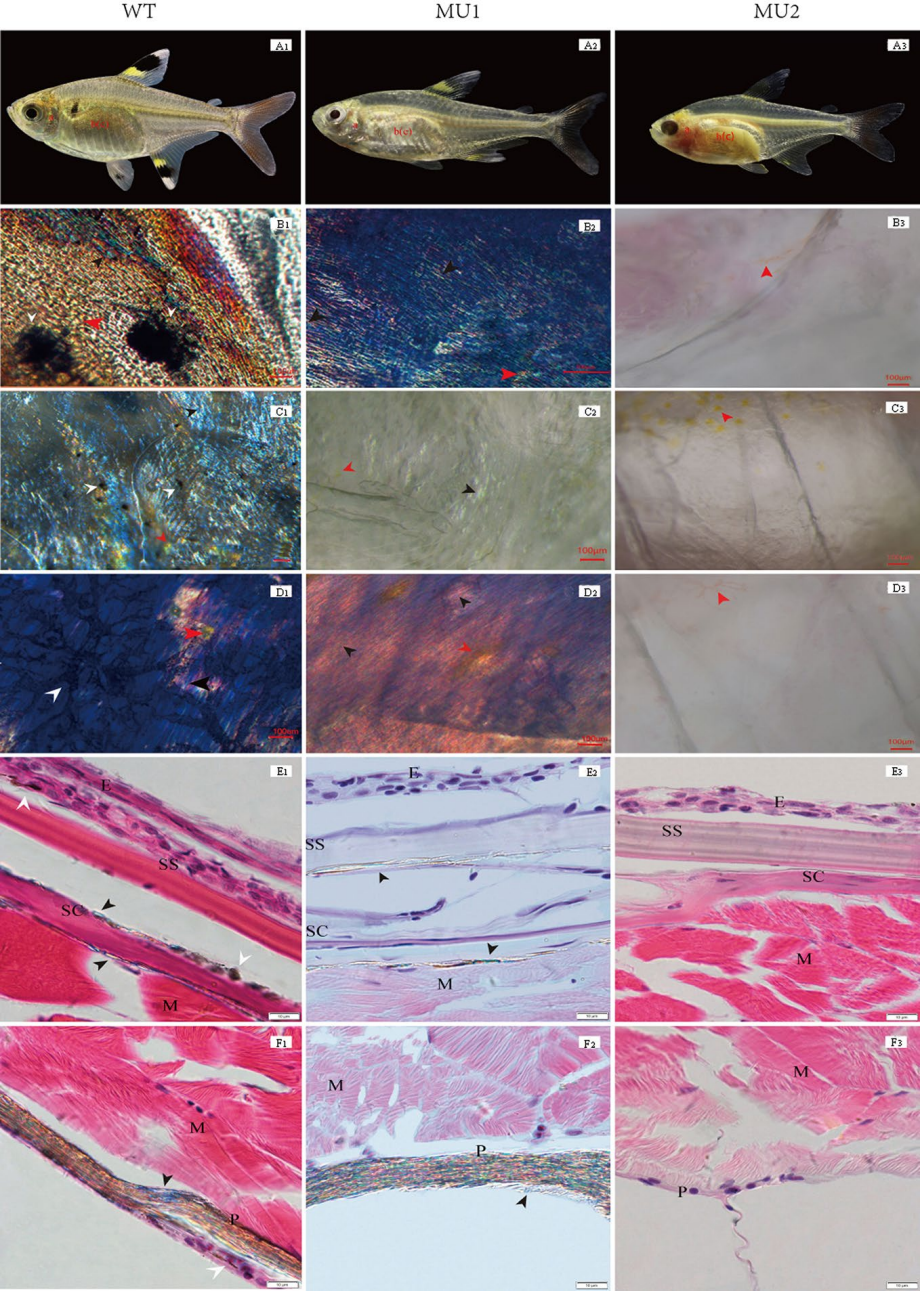
Morphological and histological observation of different body color phenotypes in *Pristella maxillaris*. The top row of fish: wild-type (WT, **A**1), Mutant I (Mu1, **A**2) and Mutant II (Mu2, **A**3). **B**-**D**: Morphological observation of pigment cells in tissues by temporary mount. Operculum lining (**B**1, **B**2, **B**3); skin (**C**1, **C**2, **C**3); peritoneum (**D**1, **D**2, **D**3). **E-F**: Histological observation of pigment cells in skin and peritoneal tissues by paraffin section. Skin (**E**1, **E**2, **E**3); peritoneum (**F**1, **F**2, **F**3); melanophores (white arrow); iridophores (black arrow); xanthophores (red arrow). E, epidermis; SS, stratum spongiosum; SC, stratum compactum; M, muscle; P, peritoneum. Scale bar = 100 µm **(B**-**D)** or 10 µm (E-F).

Chromatophores are mainly responsible for the generation of body color and can be further subdivided based on differences in body color. The types and distribution of pigment cells significantly differed among fish exhibiting the different body color phenotypes upon morphological observation. The WT phenotype was much more common than the MU1 and MU2 phenotypes, and WT individuals showed a black and gray body color (**Figure 1 A1**). The MU1 fish were translucent and showed a silvery-white body color (**Figure 1 A2**), and the MU2 individuals were completely transparent with clearly visible gill filaments and visceral tissues (**Figure 1 A3**).

Pigment cell types are easily identified due to their colors and shapes. Three types of pigment cells, i.e., melanophores, xanthophores, and iridophores, were observed in the WT operculum lining, skin, and peritoneum (**Figures 1 B1, C1, D1**). Nevertheless, in the MU1 fish, melanophores were missing in the operculum lining, skin, and peritoneal tissues, which contained only two types of pigment cells, iridophores and xanthophores (**Figures 1 B2, C2, D2**). Without melanophores and iridophores, the MU2 individuals were fully transparent (**Figures 1 B3, C3, D3**). The histological observation was used to compare the skin and peritoneum of fish exhibiting three different phenotypes of *P. maxillaris*. WT fish had many melanophores and iridophores in their skin and peritoneum (**Figures 1 E1, F1**). However, MU1 fish had many iridophores in their skin and peritoneum but had no melanophores, which was different from those in WT fish (**Figures 1 E2, F2**). In addition, melanophores and iridophores were not observed in MU2 fish (**Figures 1 E3, F3**).

### Sequencing and Assembly of the *P. maxillaris* Transcriptome

To better understand the genetics of the translucent body phenotypes, we conducted a comparative transcriptomic analysis among the three different phenotypes of *P. maxillaris* (WT, MU1, and MU2) using next-generation sequencing. After filtering the raw reads, 72.49 million, 71.70 million, and 74.89 million clean reads were generated from the skin and peritoneal tissues of WT, MU1, and MU2 fish, respectively. The detailed sequencing results are summarized in **Table 1**.

**Table 1:** Summary statistics of transcriptome sequencing for three different phenotypes of *Pristella maxillaris*.

Parameters	WT	MU1	MU2
Total raw reads	74349052	72769308	75931960
Total clean reads	72492404	71695684	74893432
Error (%)	0.02 %	0.02 %	0.02 %
Q20 percentage	97.25 %	97.62 %	97.46 %
Q30 percentage	92.66 %	93.53 %	93.20 %
N percentage	0.00 %	0.00 %	0.00 %
GC percentage	49.17 %	49.97 %	49.29 %

**Figure 2:**
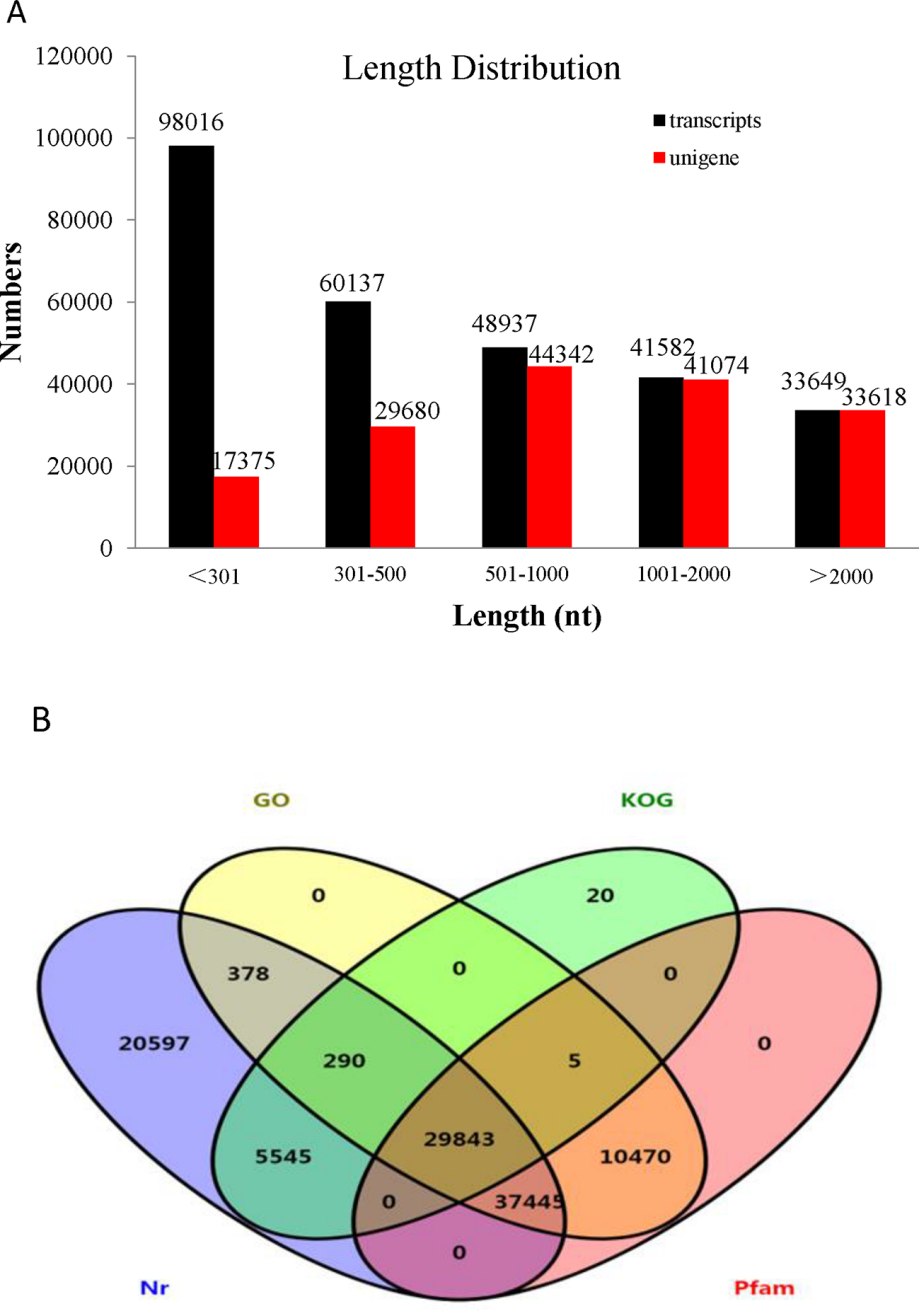
Analysis of the transcriptome data. **(A)** Length distribution of *Pristella maxillaris* unigenes and transcripts. **(B)** Venn diagram of annotation results against Nr, KOG, GO, and Pfam databases. The number in each color block indicates the number of unigenes that is annotated by single or multiple databases.

Transcriptome assemblies obtained from the three different transcriptome libraries were pooled and used to assemble full-length transcripts without reference genomes by Trinity software. After the elimination of redundant transcripts, 166,089 unigenes were acquired that ranged from 201 to 50,278 base pairs (bp) in length with a mean length of 1,293 bp and an N50 of 2,018 bp (**Supplementary Table S2**). In addition, the size distribution of the transcripts and unigenes is presented in **Figure 2A**. The unigenes provided the basis for the gene expression analysis in the skin and peritoneal tissues from fish exhibiting the three phenotypes of *P. maxillaris*.

### Annotation and Functional Classification

**Figure 3:**
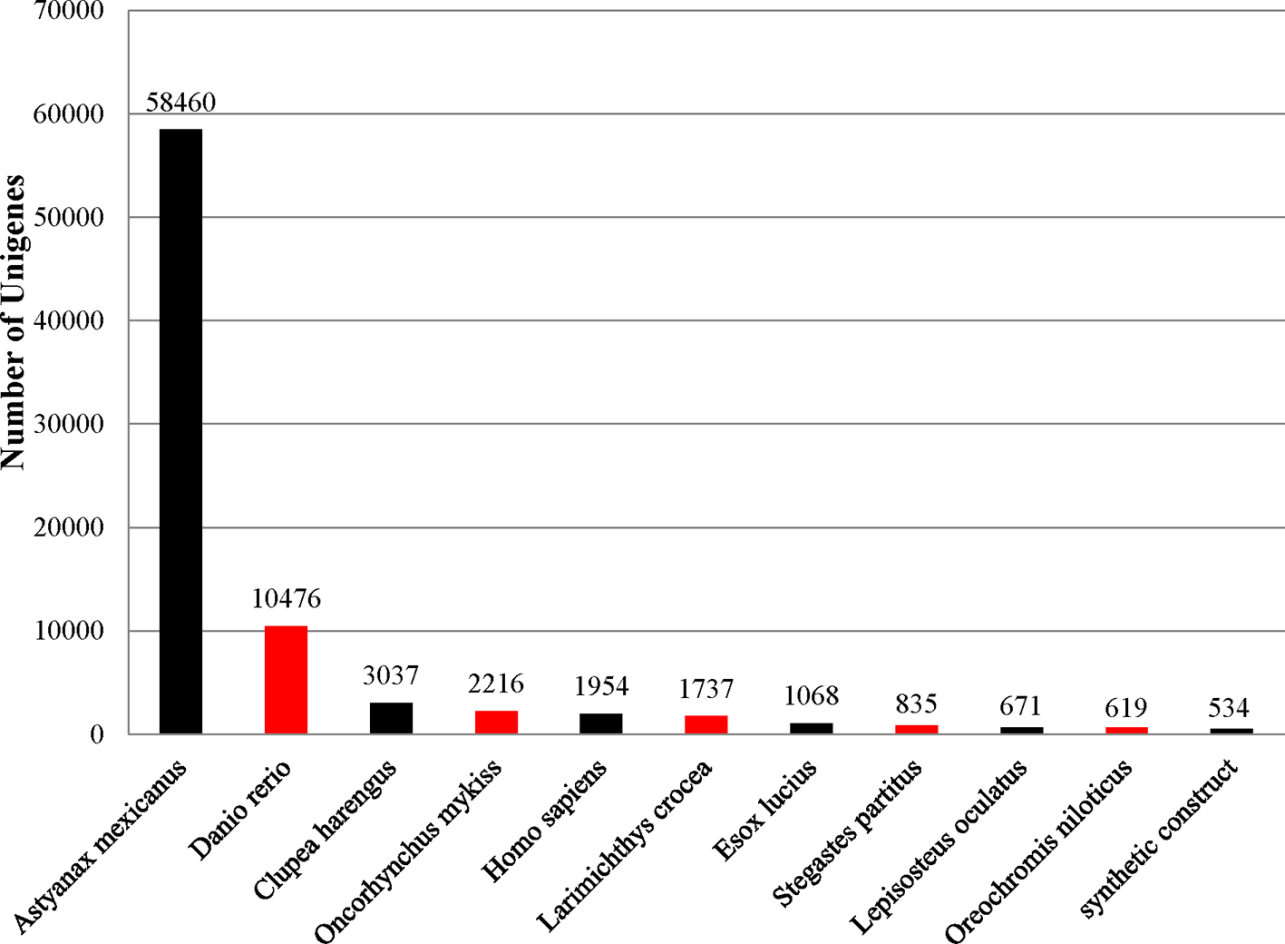
Top-hit species distribution for sequences from *Pristella maxillaris* submitted BLASTX against the NCBI-Nr database.

To identify functional information about the assembled unigenes, all of 166,089 unigenes sequences were used to search against four public databases: the NCBI non-redundant protein (Nr) database, euKaryotic Ortholog Groups (KOG), the Gene Ontology (GO) database, and the Protein family (Pfam) database. The annotation results are demonstrated by a Venn diagram (**Figure 2B**). Approximately 94,111 (56.61%), 130,854 (78.78%), 35,703 (21.49%), 78,431 (47.22%), and 77,763 (46.82%) of the unigenes were identified from the Nr, Nt, KOG, KEGG, and Pfam databases, respectively (**Supplementary Table S3**). Furthermore, 139,108 (83.75%) unigenes were simultaneously annotated in more than one database. Analysis of the BLASTX top-hit species distribution showed that 58,760 (35.20%) unigenes were similar to the *Astyanax mexicanus* sequence, 10,476 (6.31%) were similar to the *Danio rerio* sequence, 3,037 (1.83%) were similar to the *Clupea harengus* sequence, and 2,216 (1.33%) were similar to the *Oncorhynchus mykiss* sequence (**Figure 3**).

### Recognition of DEGs in Three Different *P. maxillaris* Phenotypes

**Figure 4:**
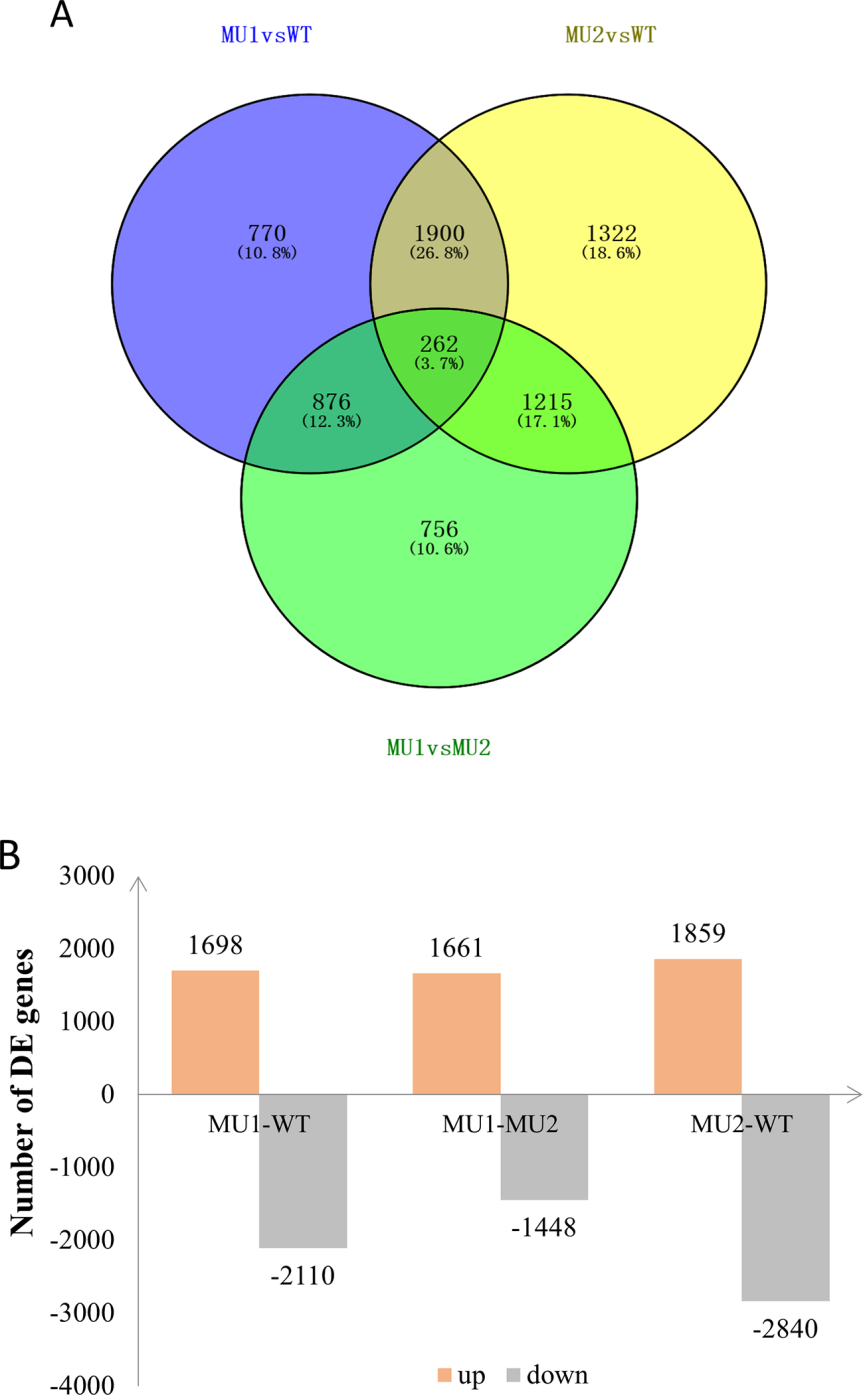
Differentially expressed genes (DEGs) in the three different phenotypes of *Pristella maxillaris* by Venn diagram **(A)** or bar chart **(B)**. The number of DEGs identified in each library contrast applying a threshold of the ratio change and a *p* value of < 0.05. The light red/gray column represents genes up-/down-regulated in different color *P. maxillaris*.

To reveal differences in the chromatophores of the skin and peritoneal tissues in *P. maxillaris* with different phenotypes, we performed a comparative analysis of the three transcriptomes. Based on criteria in which a q value < 0.005 and |Log2(fold change)| > 1 indicate a DEG, we identified 3,808 DEGs between MU1 and WT fish, of which 1,698 were upregulated and 2,110 were downregulated. We also identified 4,699 DEGs between MU2 and WT fish, including 1,859 upregulated genes and 2,840 downregulated genes. In addition, 3,109 DEGs were detected between MU1 and MU2 fish, of which 1,661 were upregulated and 1,448 were downregulated (**Figure 4**).

### Functional Enrichment of Differentially Expressed Genes

**Figure 5:**
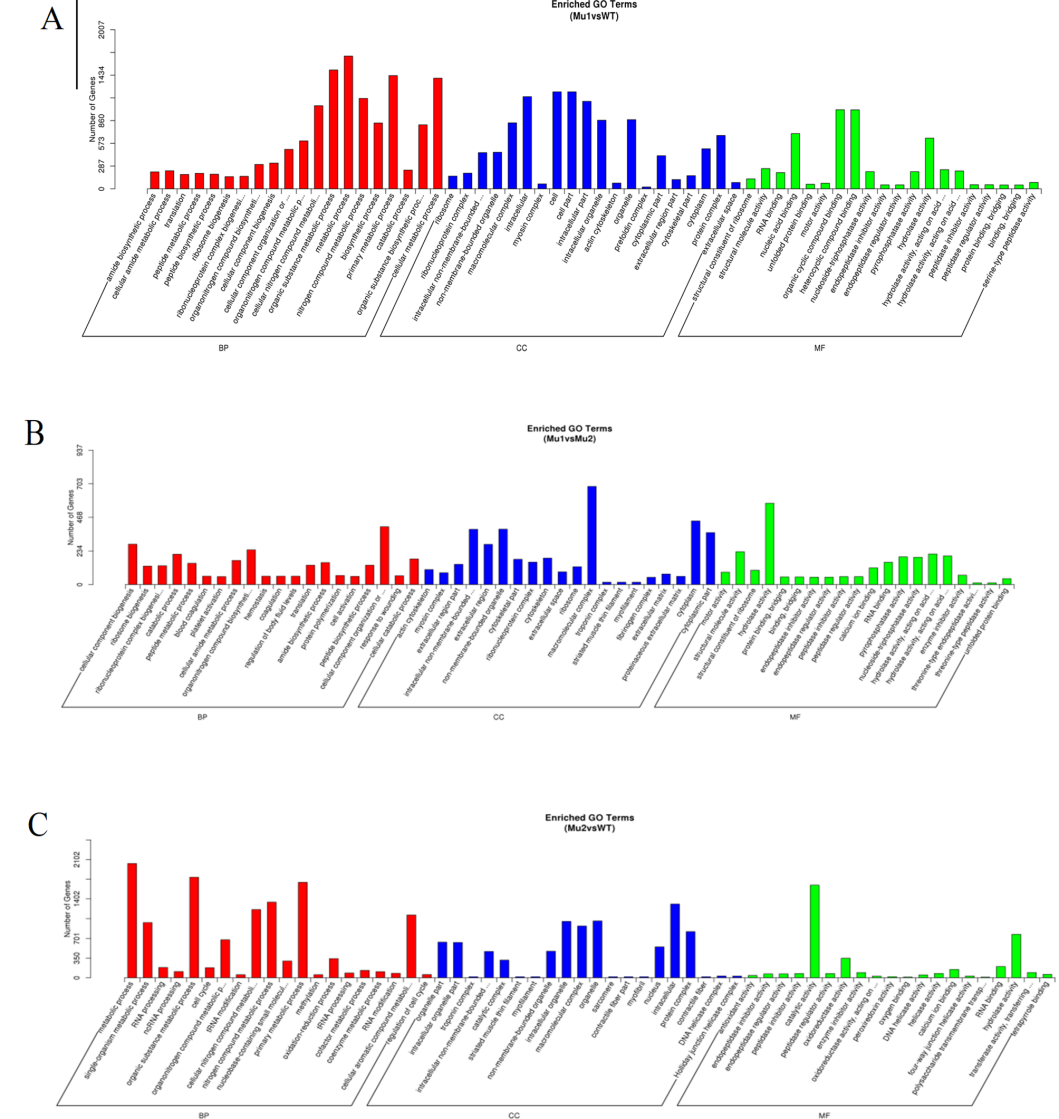
Gene Ontology (GO) functional classification of DEGs in MU1 vs. WT **(A)**, MU2 *vs.* WT **(B)**, and MU1 *vs.* MU2 **(C)**.

By further analysis of GO term enrichment and the KEGG pathways of the DEGs, all the DEGs were classified into different gene ontologies and pathways. After GO annotation, the DEGs between the MU1 and WT, MU2 and WT, and MU1 and MU2 fish were classified into 58 GO terms, 60 GO terms, and 61 GO terms, respectively. Most DEGs were mainly enriched in the following pigmentation-related terms: melanosome, pigment catabolic process, tyrosine biosynthetic process, tRNA (guanine) methyltransferase activity, pigment metabolic process, tyrosine metabolic process, calcium ion transport, purine nucleoside metabolic process, activation of MAPK activity, purine ribonucleotide binding, regulation of Wnt signaling pathway, tricarboxylic acid cycle, and purine-containing compound biosynthetic process. The DEGs were classified into biological processes, cellular components, and molecular functions (as shown in **Figure 5**). The cellular processes and metabolic processes were the two largest categories within the biological processes; the two largest molecular function categories were binding and catalytic activity; the most abundant categories were cell and intracellular for the cellular components.

The DEGs in the skin and peritoneal tissues in three different phenotypes of *P. maxillaris* were annotated in the KEGG database. The DEGs between the MU1 and WT, MU2 and WT, and MU1 and MU2 fish participated in 20 pathways (**Table 2**) and were significantly enriched. Among the DEGs between the MU1 and WT fish involved in these 20 pathways, most of the DEGs involved in DNA replication, mismatch repair, nucleotide excision repair, oxidative phosphorylation, and the citrate cycle were downregulated in the MU1 fish compared with their expression in the WT fish (**Table 2a**). At the same time, some of the DEGs involved in tyrosine metabolism and melanogenesis were upregulated in the WT fish compared to their expression in the MU1 fish. The DEGs between the MU2 and WT fish were significantly enriched in some metabolic pathways, including purine metabolism, nucleotide excision repair, the pentose phosphate pathway, glycolysis/gluconeogenesis, mismatch repair, oxidative phosphorylation, tyrosine metabolism, and the melanogenesis pathway (**Table 2b**). Besides, some DEGs involved in purine metabolism, the pentose phosphate pathway, glycolysis/gluconeogenesis, and tyrosine metabolism were downregulated in the MU2 fish compared with the WT fish. In addition, the ECM receptor interaction, protein digestion and absorption, proteasome, glycolysis/gluconeogenesis, DNA replication, and purine metabolism terms were significantly enriched in most DEGs between the MU1 and MU2 fish (**Table 2c**). Meanwhile, some DEGs involved in glycolysis/gluconeogenesis, DNA replication, and purine metabolism were upregulated in the MU1 fish compared with the MU2 fish.

**Table 2:** Kyoto Encyclopedia of Genes and Genomes (KEGG) functional analysis of DEGs in MU1 vs. WT (a), MU2 vs. WT (b), and MU1 vs. MU2 (c).

Pathway (MU1*vs*WT, a)	DEGs*with Pathway	q-Value	Pathway ID
Pathway (MU2*vs*WT, b)	DEGs*with Pathway	q-Value	Pathway ID
Pathway (MU1*vs*MU2, c)	DEGs*with Pathway	q-Value	Pathway ID
Ribosome	109	1.69E-31	ko03010
DNA replication	20	6.13E-05	ko03030
Mismatch repair	12	0.003566778	ko03430
Systemic lupus erythematosus	38	1.66E-10	ko05322
Nucleotide excision repair	19	4.52E-05	ko03420
Staphylococcus aureus infection	24	0.000216615	ko05150
Glutathione metabolism	20	0.00019	ko00480
RNA degradation	28	2.77E-05	ko03018
Pyrimidine metabolism	42	4.45E-07	ko00240
Legionellosis	23	0.00018	ko05134
Antigen processing and presentation	32	3.81E-05	ko04612
Cell cycle	45	2.10E-06	ko04110
Oxidative phosphorylation	38	1.26E-05	ko00190
Parkinson’s disease	44	5.55E-06	ko05012
RNA transport	59	7.41E-07	ko03013
Purine metabolism	51	6.54E-05	ko00230
MAPK signaling pathway	30	0.997786982	ko04010
cAMP signaling pathway	22	0.996297986	ko04024
Melanogenesis	44	0.910326122	ko04916
Tyrosine metabolism	4	0.50194862	ko00350
Pyrimidine metabolism	65	1.25E-11	ko00240
DNA replication	33	6.91E-11	ko03030
Cell cycle	69	6.91E-11	ko04110
Systemic lupus erythematosus	44	2.56E-09	ko05322
Purine metabolism	71	1.92E-06	ko00320
Nucleotide excision repair	27	4.80E-06	ko03420
Spliceosome	59	8.56E-05	ko03040
Pentose phosphate pathway	19	0.000103465	ko00030
Mismatch repair	16	0.000320844	ko03430
RNA polymerase	17	0.000522379	ko03020
Staphylococcus aureus infection	26	0.000547331	ko05150
RNA degradation	32	0.000635092	ko03018
Glycine, serine and threonine metabolism	18	0.001075308	ko00260
Cardiac muscle contraction	43	0.001075308	ko04260
ECM-receptor interaction	43	0.002053638	ko04512
Parkinson’s disease	46	0.002072794	ko05012
Oxidative phosphorylation	39	0.004989829	ko00190
Glycolysis / Gluconeogenesis	26	0.008887468	ko00010
Tyrosine metabolism	8	0.090654	ko00350
Melanogenesis	22	0.822218	ko04916
Ribosome	89	2.75E-25	ko03010
ECM-receptor interaction	61	2.63E-11	ko04512
Protein digestion and absorption	75	5.32E-18	ko04974
Tight junction	93	8.50E-13	ko04530
Pathogenic Escherichia coli infection	42	1.08E-07	ko05130
Proteasome	20	2.32E-07	ko03050
Glycolysis / Gluconeogenesis	29	2.90E-07	ko00010
Hypertrophic cardiomyopathy (HCM)	44	4.50E-06	ko05410
DNA replication	16	4.74E-05	ko03030
PI3K-Akt signaling pathway	89	0.000509	ko04151
Focal adhesion	87	1.08E-05	ko04510
Dilated cardiomyopathy (DCM)	44	1.20E-05	ko05414
Systemic lupus erythematosus	25	3.36E-05	ko05322
Metabolism of xenobiotics by cytochrome P450	12	0.047531	ko00980
Purine metabolism	33	0.080042	ko00230
Pentose pphosphate athway	11	0.001984	ko00030
Spliceosome	43	6.88E-05	ko03040
Cardiac muscle contraction	33	0.000210	ko04260
Antigen processing and presentation	27	0.000417	ko04612
Arginine biosynthesis	13	0.000454	ko00220

*DEGs, differentially expressed genes, which was identified by the DEGseq package. DEGs between the two samples were selected with the following filter criteria: log2 transcript abundance ratio ≥1 and FDR (false discovery ratio) ≤ 0.001.

### Candidate Genes Related to Chromatophores

According to zebrafish ensemble database (http://asia.ensembl.org/Daniorerio/Info/Index), 97 genes were annotated in the pigmentation category. After a BLAST search with the 97 pigmentation-related genes, a total of approximately 40 melanophore- and iridophore-related genes were detected in the *P. maxillaris* skin and peritoneal tissue. Considering the FPKM (expected number of fragments per kilobase of transcript sequence per millions base pairs sequenced) of these genes, we found 14 genes that enriched the tyrosine metabolism and melanogenesis pathways that were expressed at a significantly higher level in WT fish than in MU1 and MU2 fish and nine genes with significantly higher expression in WT fish (**Table 3**). Among the DEGs, the *protein Wnt-8a* (*wnt8*), *frizzled 2* (*fzd2*), *agouti-signaling protein* (*asip*), *cyclic AMP-responsive element-binding protein 3-like protein 4* (*creb*), and *dual specificity mitogen-activated protein kinase 2-like* (*map2k2*) were found to be the most highly expressed genes in the WT fish, followed by the *alcohol dehydrogenase 6-like* (*adh6*), *glutathione S-transferase* (*gst*), *guanine nucleotide-binding protein* (*gnai*), and *calmodulin-like* (cam) genes.

**Table 3:** KEGG pathway analysis of positively selected genes involved in melanophores in *P. maxillari* *s*.

Gene ID	Gene name	Discription	KEGG pathway
Cluster-7922.70780	*adh6*	alcohol dehydrogenase 6-like	Tyrosine metabolism
Cluster-7922.15576	*gst*	glutathione S-transferase	Drug metabolism - cytochrome P450
Cluster-7922.27281	*wnt8*	protein Wnt-8a	Wnt/β-catenin signaling pathway
Cluster-7922.40920	*fzd2*	frizzled 2	Melanogenesis
Cluster-7922.92050	*asip*	agouti-signaling protein	Melanogenesis
Cluster-7922.45484	*creb3*	cyclic AMP-responsive element-binding protein 3-like protein 4,	Melanogenesis
Cluster-7922.33657	*map2k2*	dual specificity mitogen-activated protein kinase kinase 2-like	MAPK signaling pathway
Cluster-7922.68121	*gnai*	guanine nucleotide-binding protein G(o) subunit alpha isoform X1	Melanogenesis
Cluster-7922.65792	*cam*	calmodulin-like	Melanogenesis
Cluster-7922.58712	*bad*	bcl2 antagonist of cell death-like isoform X1	Melanoma
Cluster-7922.100710	*cdk4*	cyclin-dependent kinase 4	Melanoma
Cluster-7922.81890	*dvl2*	segment polarity protein dishevelled homolog	Melanogenesis
Cluster-7922.62453	*kras*	GTPase KRas isoform X1	Melanogenesis
Cluster-7922.78643	*camk2*	calcium/calmodulin-dependent protein kinase type II	Melanogenesis

Through GO term and KEGG pathway analyses of the significant DEGs, a total of 26 DEGs were involved in glycolysis/gluconeogenesis, purine metabolism, and the pentose phosphate pathway, which play an important role in iridophore development. Fifteen of the 26 genes are detailed in **Table 4**. Interestingly, seven crucial genes were identified in these pathways, including *trifunctional purine biosynthetic protein adenosine-3* (*gart*), *hypoxanthine guanine phosphoribosyl transferase* (*hprt*), *beta-enolase* (*eno*), *nucleoside diphosphate kinase-like* (*ndk*), *guanylate kinase isoform X1* (*guk1a*), *phosphoglycerate mutase 1-like* (*pgam1*), and *6-phosphofructokinase, muscle type* (*pfka*), which were significantly upregulated in the WT and MU1 fish but significantly downregulated in the MU2 fish (*p* value ≤ 0.005). In contrast, the expression of the *bifunctional purine biosynthesis protein* (*pur9*) and *L-lactate dehydrogenase B-A chain* (*ldh*) genes did not differ among the three different translucent body phenotypes.

**Table 4:** KEGG pathway analysis of positively selected genes involved in iridophores metabolism in *P. maxillaris*.

Gene ID	Gene name	Discription	KEGG Pathway
Cluster-7922.60112	*ldh*	L-lactate dehydrogenase B-A chain	Glycolytic pathway
Cluster-7922.64440	*pk*	pyruvate kinase PKM isoform X1	Purine metabolism
Cluster-7922.57158	*eno*	beta-enolase	Glycolysis / Gluconeogenesis
Cluster-7922.63283	*aldo*	fructose-bisphosphate aldolase C-B-like	Glycolysis / Gluconeogenesis
Cluster-7922.61593	*pur9*	bifunctional purine biosynthesis protein	Purine metabolism
Cluster-7922.45519	*hprt*	hypoxanthine-guanine phosphoribosyltransferase	Purine metabolism
Cluster-7922.59115	*pgam1*	phosphoglycerate mutase 1-like	Glycolysis / Gluconeogenesis
Cluster-7922.51649	*guk1a*	guanylate kinase isoform X1	Purine metabolism
Cluster-7922.49116	*pfka*	6-phosphofructokinase, muscle type	pentose phosphate pathway
Cluster-7922.79755	*tktl*	transketolase-like protein 2	pentose phosphate pathway
Cluster-7922.64418	*taldo*	transaldolase	pentose phosphate pathway
Cluster-7922.34530	*pgd*	6-phosphogluconate dehydrogenase, decarboxylating-like	pentose phosphate pathway
Cluster-7922.52874	*impdh2*	monophosphate dehydrogenase 2	Purine metabolism
Cluster-7922.21114	*ndk*	nucleoside diphosphate kinase-like isoform X1	Purine metabolism
Cluster-7922.62678	*gart*	trifunctional purine biosynthetic protein adenosine-3	Purine metabolism

**Figure 6:**
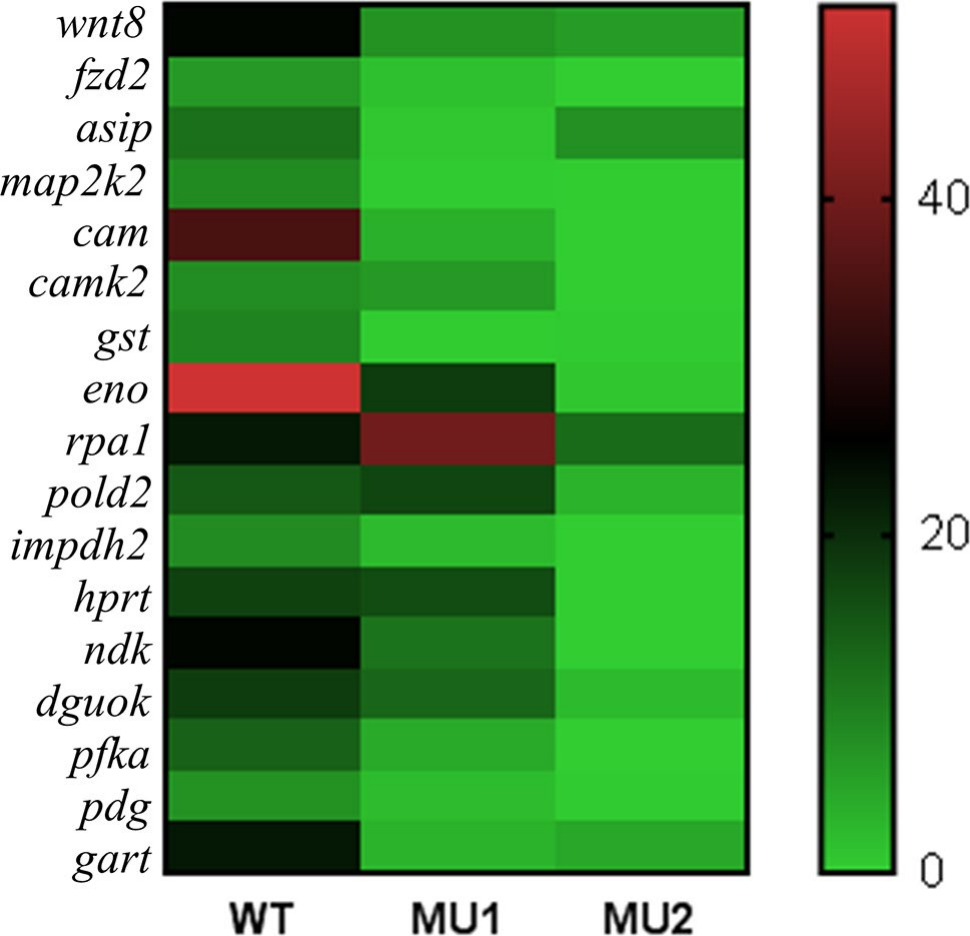
Heat map showing the expressions of selected DEGs in three body color phenotypes of *P. maxillaris*.

The GO term enrichment and KEGG pathway analyses of the DEGs identified candidate genes that could regulate melanophore or iridophore development. We also analyzed the mRNA expression of 17 candidate genes (**Figure 6**). Some genes, such as *wnt8*, *fzd2*, *map2k2*, *cam*, *creb*, and *gst*, were significantly upregulated in WT fish compared with the MU1 and MU2 fish. However, the mRNA expression levels of some iridophore-related genes (*pfka*, *eno*, *gart*, and *hprt*) were markedly lower in the MU2 fish than in the WT and MU1 fish.

### Confirmation of DEGs Identified with RNA-Seq by Quantitative Real-Time PCR

**Figure 7:**
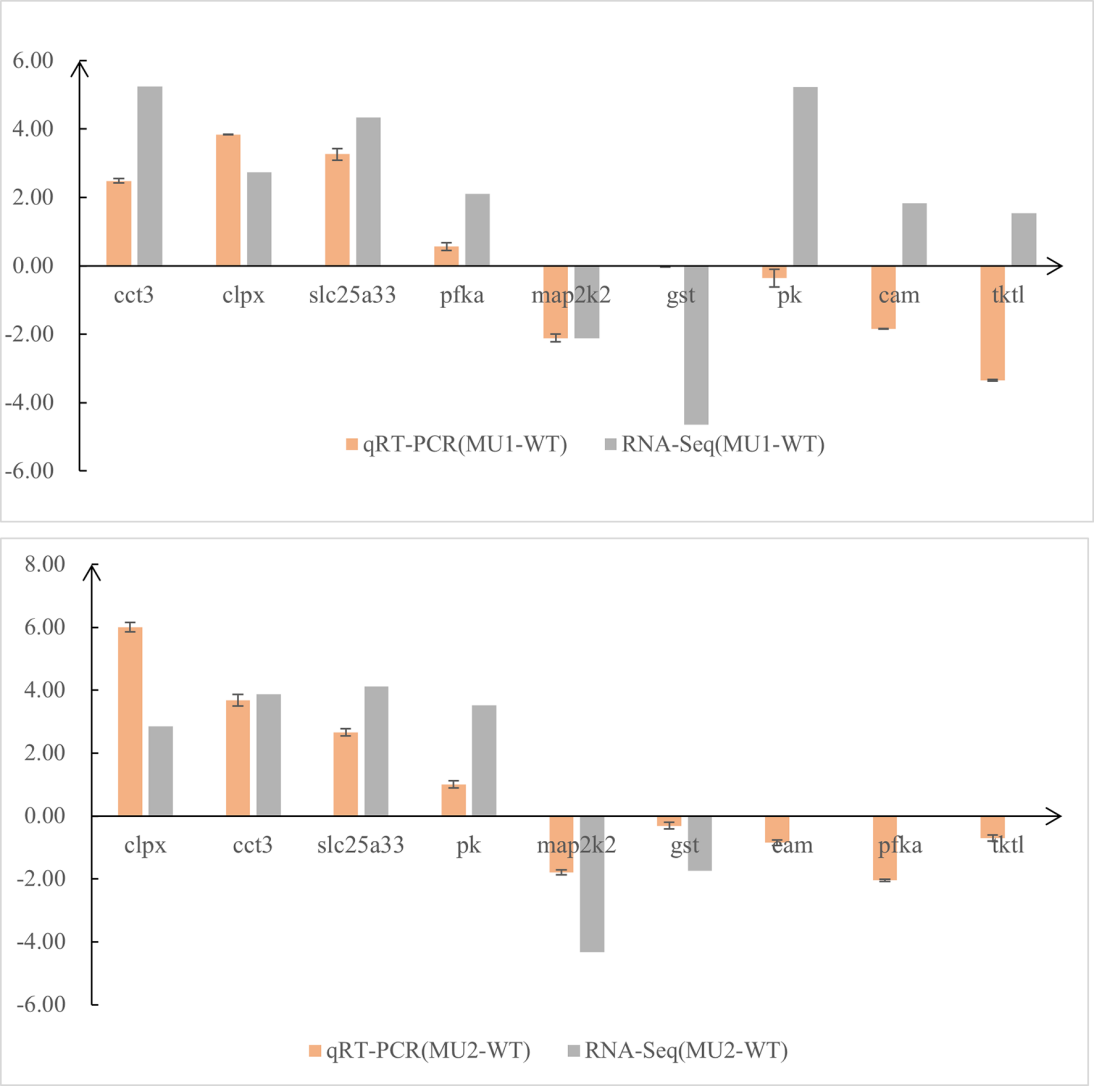
Comparison of gene expression patterns obtained using RNA-Seq and qRT-PCR. Log-fold changes are expressed as the ratio of gene expression after normalization to *gapdh*.

To test the DEGs identified by comparative transcriptomic analysis, we selected nine genes from the three comparative groups and the *gapdh* gene for qRT-PCR confirmation. The quantitative real-time PCR (qRT-PCR) expression patterns of 6 of 9 randomly selected DEGs that were related to pigment biosynthesis agreed with the results from RNA-Seq analysis, except for the *pk* (*pyruvate kinase*), *cam*, and *transketolase-like protein 2* (*tktl*) genes (**Figure 7**). Therefore, the expression patterns of the selected genes determined by qRT-PCR were nearly in accordance with the RNA-Seq data. Combining the qRT-PCR and RNA-Seq results, we found that melanin-related genes were more highly expressed in the skin and peritoneal tissues of WT fish than in mutant fish, whereas guanine-related genes were more highly expressed in the WT and MU1 fish than in the MU2 fish. However, the melanin-related genes and guanine-related genes were expressed at lower levels in MU2 fish compared with the WT and MU1 fish.

## Discussion

Animal coloration plays an important role in ecological interactions, species recognition, and even protecting the organism from ultraviolet radiation damage (Muske and Fernald, 1987; Lowe and Goodman-Lowe, 1996; Fujimura et al., 2009; Sefc et al., 2014). Diverse body coloration is mainly controlled by the development and location of pigment cells. The variety and number of pigment cells affect animal body transparency (Nilsson Skold et al., 2010; Croucher et al., 2013; Nilsson Skold et al., 2013). In this study, we observed the different morphologies of transparent body parts from three different phenotypes of *P. maxillaris*, and revealed significant differences in the types and distributions of pigment cells by microscopic observation as performed in other literatures (Kelsh, 2004; Darias et al., 2013; Zhang et al., 2015; Zhang et al., 2017). Moreover, we also found that changes in the type and number of pigment cells led to different phenotypes and increased the transparency of the *P. maxillaris* body. Extensive research has been performed on this topic, and many species of fish have been shown to change their internal color due to responsive peritoneal chromatophores, in which the degree of this response was correlated with the level of body transparency (Parichy, 2007; Nilsson Skold et al., 2010). Meanwhile, Krauss indicated that the inner organs were observed through the skin due to the loss of iridophores (Krauss et al., 2013). Our results suggested that the loss of melanophores and iridophores resulted in changing of body color from gray to transparent during *P. maxillaris* reproduction. In the study, xanthophores were not observed in the different phenotypes fish by histological method. The reason may be that the inclusions of xanthophores are fat-soluble carotenoids and water-soluble dinidine (Hirata et al., 2003), which are easy to dissolve during dehydration and repeated washing. In future studies, we will try to use the other method to observe the xanthophores.

Genetic factors, which are the major determinants of animal body color, influence the production and distribution of pigment cells. In recent years, the mechanism of body color formation in fish has received attentions such as transcriptome analyses of model or economic fish including zebrafish (Irion et al., 2016), crucian carp (Zhang et al., 2017), and the common carp (Wang et al., 2014). In this study, we used Illumina sequencing technology to examine the skin and peritoneal tissues from fish exhibiting three phenotypes of *P. maxillaris* at the transcriptome level and found many DEGs associated with pigmentation. The identified DEGs among the three phenotypes could help us understand the molecular mechanism and provide valuable genetic information to explore pigmentation in the future.

The GO enrichment analysis of the DEGs revealed that variations in pigmentation are related to cellular components and biological processes. Most of the clustered groups of DEGs were consistent with those identified in previous works with fish such as zebrafish (Higdon et al., 2013), Midas cichlids (Henning et al., 2013), crucian carp (Zhang et al., 2017), and common carp (Jiang et al., 2014; Li et al., 2015). Interestingly, we found that most of the genes downregulated in the MU2 fish compared to their expression in the WT fish were enriched in GO terms related to the pigment metabolic process, including the tyrosine metabolic process, the activation of MAPK activity, tyrosine 3-monooxygenase activity, the pigment catabolic process, the purine-containing compound biosynthetic process, tRNA (guanine) methyltransferase activity, and the purine nucleobase biosynthetic process. In addition, most of the genes downregulated in the MU1 fish compared with their expression in the WT fish were enriched in the tyrosine metabolic process, activation of MAPK activity, tyrosine 3-monooxygenase activity, and pigment biosynthetic process GO terms.

The KEGG pathway analysis showed that some DEGs were associated with pigmentation-related pathways. In our study, some DEGs between the MU1 and WT fish were enriched in the tyrosine metabolism, melanogenesis, cAMP signaling, and Wnt or MAPK signaling pathways. Both the cAMP and MAPK signaling pathways are involved in melanophore development in vertebrates (Zhang et al., 2015; Zhang et al., 2017). The DEGs in *P. maxillaris* were likely involved in melanin synthesis. Meanwhile, we found that some DEGs between the MU2 and WT fish were enriched in the glycolysis/gluconeogenesis, purine metabolism, and pentose phosphate pathway terms. The identification of genes enriched in these pigmentation-related terms and pathways are informative, and these genes are worth further study.

**Figure 8:**
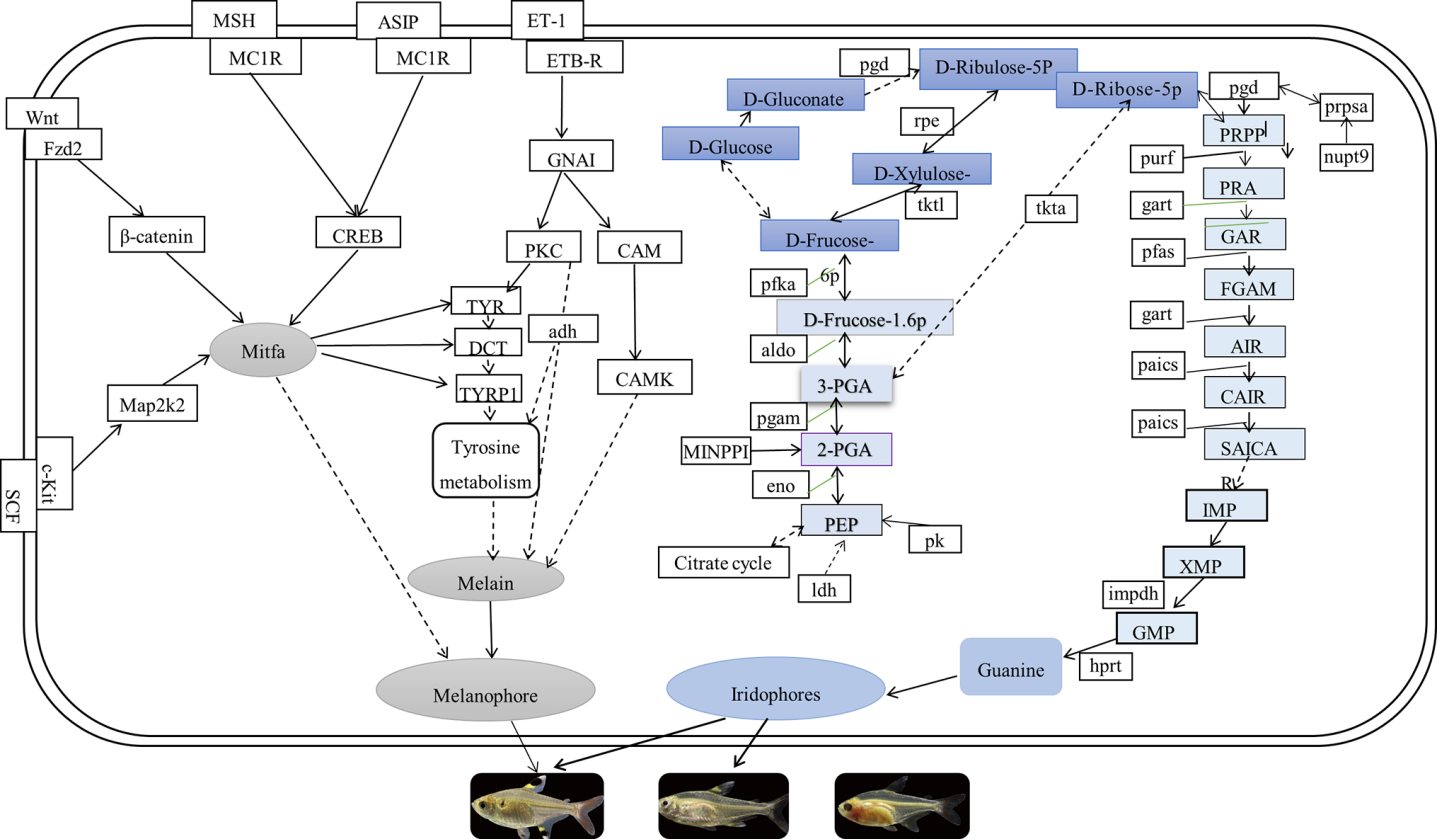
The putative genes and pathways involved in pigmentation process of three phenotypic of *Pristella maxillaris* based on the present study.

In this study, comparing known pigmentation genes with identified genes by the current transcriptome data, we found many of  the pigmentation genes and pathways in *P. maxillaris*. The putative genes and pathways involved in the three body transparency phenotypes that are related to the pigmentation process are shown in **Figure 8**. We found that the mRNA expression levels of *wnt8*, *fzd2*, *map2k2*, *creb*, *asip*, and *cam* were downregulated in the skin and peritoneal tissues of MU2 fish compared to the WT fish. Several studies have reported that the Wnt signaling pathway participates in the synthesis of melanogenesis in teleost fishes, as well as in mammals (Fujimura et al., 2009; Xing et al., 2011; Zhang et al., 2015). *Wnt8*, a noncanonical Wnt protein family gene, was found in the matrix and precortical cells in the hair follicles of mice (Yamaguchi et al., 1999; Croucher et al., 2013). Interestingly, *wnt8* was expressed at lower levels in the MU1 and MU2 fish than in the WT fish. The wnt8 can bind with *fzd2* to promote the production of guanine-binding protein (Go/Gq), which in turn promotes the expression of β-catenin, thereby inducing the expression of *mitf* (*melanocyte inducing transcription factor*). The *mitf* is a key regulatory gene in the melanophore lineage (Levy et al., 2006; Zeng et al., 2015). Some transcription factors, such as β-catenin and sox10 (*SRY-box containing gene 10*), have been reported to act on the promoter region of *mitf*, which promotes the expression of *mitf* (Sakai et al., 1997; Zhang et al., 2017). In addition, *mitf* directly regulates the expression of multiple genes (*tyr*, *tyrp1*, and *dct* [*dopachrome tautomerase*]) that are necessary for the survival and proliferation of melanophores (Opdecamp et al., 1997; Cheli et al., 2010) and are responsible for the synthesis of melanin (Li et al., 2012). These results show that *wnt8*, *fzd2*, and* β-catenin* might play important roles in the body transparency phenotypes of *P. maxillaris*.

We also found that the dual specificity *map2k2* and *cam* genes were significantly upregulated in the WT fish compared to the MU1 and MU2 fish. *Cam* is activated by cytoplasmic Ca^2+^, which is released from the endoplasmic reticulum to assist protein kinase C (Ma et al., 2014). Additionally, protein kinase C can expand the promotion of melanin synthesis by protein kinase A by upregulating the *mitf* gene (Park et al., 2006). Another gene, *map2k2*, encodes an important enzyme in the MAPK signaling pathway that can activate *mitf*, increase *mitf* expression, and then stimulate the synthesis of melanin (Levy et al., 2006). In addition, the mRNA expression level of *asip* decreased as the *P. maxillaris* body color changed from gray to transparent. Asip was an endogenous antagonist of alpha-melanocyte stimulating hormone (α-MSH). The α-MSH causes an increase in tyrosinase activity, and α-MSH could activate the *melanocortin 1 receptor* (*mc1r*), a key gene in melanogenesis in animals, resulting in increased cAMP levels. Consequently, the melanin biosynthesis process is triggered (Voisey et al., 2001; Henning et al., 2010; Zhang et al., 2017). On the contrary, Asip can block melanin synthesis by competing with α-MSH in binding to the *mc1r* gene (Sakai et al., 1997; Zhang et al., 2017). Histological assessment revealed that MU1 and MU2 do not possess melanophores. In addition, the results revealed that *asip* might not inhibit melanin synthesis in MU1 and MU2 fish. We also found that the *creb* gene was significantly upregulated in the WT fish compared to its expression in MU1 and MU2 fish. It has been reported that *mc1r* activates the *creb*, and its cascade involves the upregulation of the expression of *mitf*, which binds and activates melanogenic gene promoters to increase their expression, resulting in increased melanin synthesis (Busca and Ballotti, 2000). Therefore, it was again confirmed that *creb* might play a key role in melanin production.

We also discovered some DEGs between group of MU2 and WT and group of MU1 and MU2 fishes involved in purine metabolism, glycolysis/gluconeogenesis, the citric acid cycle, and the pentose phosphate pathway such as the β-*eno*, *gart*, *aldo* (*fructose-bisphosphate aldolase C-B-like*), *ldh*, and *hprt* genes. These genes exhibited significantly lower expression in MU2 fish compared to the WT and MU1 fish, followed by *guk1a* and *pfka*, which implied the participation of these pathways in body coloration in MU2 fish. Fish skin and other tissues contained stacks of guanine plates in iridophores (Hirata et al., 2003; Hirata et al., 2005; Failde et al., 2014), and glycolysis and the citrate cycle pathway were found to be key participants in extensive guanine synthesis (Higdon et al., 2013; Irion et al., 2016). Combined with our microscopic observations, many iridophores were observed in the WT and MU1 fish, but fish with the MU2 mutation did not harbor iridophores. Thus, the increased expression of genes within these pathways might be in accordance with the increased requirement of guanine for the reflective iridophore pigment in the skin and peritoneal tissues of WT and MU1 fish.

In the purine metabolism pathway, the *gart* and *phosphoribo sylaminoimidazolesuccinocarboxamide synthase* (*paics*) genes combine into a complex that is involved in the synthesis of inosine monophosphate, a precursor of the purine nucleotides adenosine monophosphate and guanosine monophosphate. Some studies have shown that guanosine monophosphate synthase increases the number of iridophores (Ng et al., 2009). In addition, Higdon et al. (2013) illustrated that specific enzymes such as *aldo*, *eno*, *pgam1*, and *gart* could regulate guanine synthesis. These results revealed the conservation of pigmentation genes across various species in terms of their sequences and functions. However, further investigations are still needed to determine how these genes work together to regulate guanine synthesis in iridophores.

To test the reliability of the RNA-Seq data, nine genes were randomly selected for qRT-PCR, including *chaperonin containing tcp1 subunit 3* (*cct3*), *solute carrier family 25 member 33* (*slc25a33*), *glutathione s-transferase* (*gst*), and *map2k2*, and so on. The expression pattern of these pigment-specific genes by qRT-PCR coincided with the results of the RNA-Seq analysis, except for the *pk*, *cam*, and *tktl* genes. The expression levels measured using the two methods were roughly coincided, indicating the reliability of our transcriptome data. We found that body coloration differs among varieties and the distribution of chromatophores at the cellular level. Therefore, an increase in body transparency might be caused by the absence of melanophores and iridophores in *P. maxillaris*. Moreover, after analyzing the WT and two mutant transcriptomes, we found that differentially expressed candidate pigmentation genes mainly enriched pathways related to melanin and guanine synthesis. However, further work is still needed to determine how these pathways and genes regulate the development of melanophores and iridophores in the body transparency phenotypes of *P. maxillaris*.

In addition, we also found that most DEGs were enriched in ribosome-related pathways in the skin and peritoneal tissues of fish exhibiting different phenotypes of *P. maxillaris*, which indicated that ribosomes might play an important role in fish body color formation. Higdon et al. (2013) found that four of the five most highly expressed genes were encoding ribosomal proteins in the transcriptome of zebrafish pigment cells. A similar finding was also reported in the transcriptome analysis of sheep skin (*Ovis aries*) (Fan et al., 2013). Some studies have proven that highly expressed levels of ribosome protein-related genes are correlated to black coat color in mice (Skarnes et al., 2011). Combined with the transcriptome data, we noted that the ribosomal protein genes might be involved in the formation of body coloration in *P. maxillaris*. However, further studies are needed to elucidate its exact function. We also discovered that some DEGs are involved in nucleotide excision repair, mismatch repair, oxidative phosphorylation, and systemic lupus erythematosus signaling pathways. These genes were significantly downregulated in the MU1 and MU2 fish compared with their expression in WT fish, which might be related to the absence of melanocytes and iridophores. Some studies have indicated that melanin from melanocytes not only scatters and absorbs UV as a physical barrier but also protects other epidermal cells by transferring melanin and reducing DNA damage (Kobayashi et al., 1998; Brenner and Hearing, 2008). In addition, the scattered reflectors and the arbitrary orientations of iridophores reflected all wavelengths of light (Grether et al., 2004).

Taken together, we observed significant differences in the types and distribution of pigment cells in three different phenotypes of *P. maxillaris* and elucidated the potential genes and signaling pathways involved in body transparency.

## Data Availability

This manuscript contains previously unpublished data. The name of the accession number is PRJNA525550 (https://www.ncbi.nlm.nih.gov/bioproject/PRJNA525550).

## Author Contributions

FB, XY, RY, and TC contributed to the study design, the major acquisition, analysis, and interpretation of data, and drafting/revising the article. FB, ZO, and JL performed most of the laboratory work, and BT and MY assisted. FB contributed to the analysis of the data and wrote the manuscript. All authors read and approved the final manuscript.

## Funding

The Huazhong Agricultural University Scientific & Technological Self-Innovation Foundation (2662018PY083), the National Natural Science Foundation of China (31771648), and the Finance Special Fund of Ministry of Agricultural of China (Fisheries resources and environment survey in the key water areas of Tibet) supported this study.

## Conflict of Interest Statement

The authors declare that the research was conducted in the absence of any commercial or financial relationships that could be construed as a potential conflict of interest.
