# Supplementary material for: Morphological Characteristics and Comparative Transcriptome Analysis of Three Different Phenotypes of Pristella maxillaris
Source: Front Genet. 2019 Aug 2;10:698. doi: 10.3389/fgene.2019.00698 (PMC6687772; doi:10.3389/fgene.2019.00698)
Supplement: Supplementary file 1 [file Presentation_1.pptx]

## Slide 1
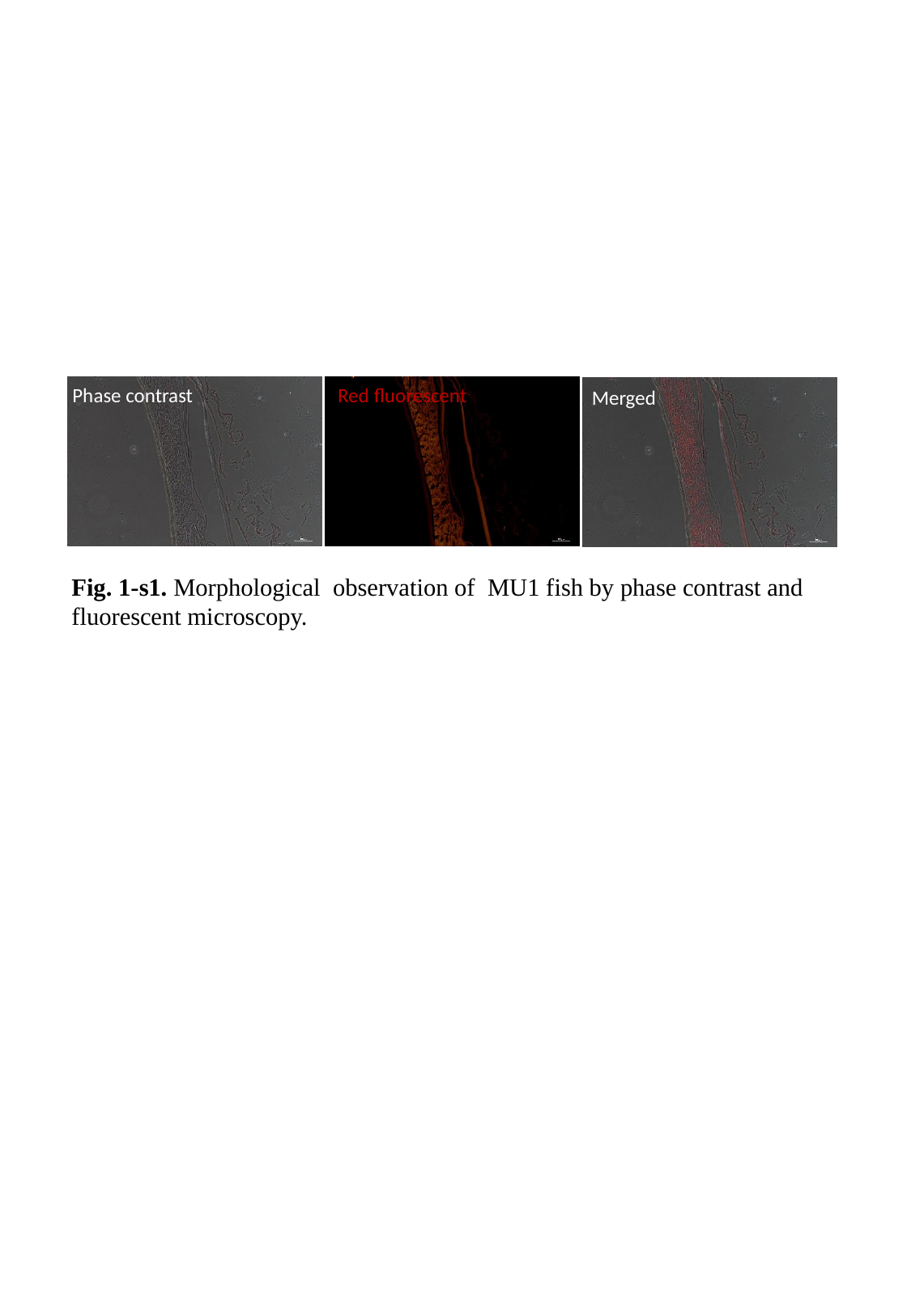

Phase contrast
Red fluorescent
Merged
Fig. 1-s1. Morphological observation of MU1 fish by phase contrast and fluorescent microscopy.

## Slide 2
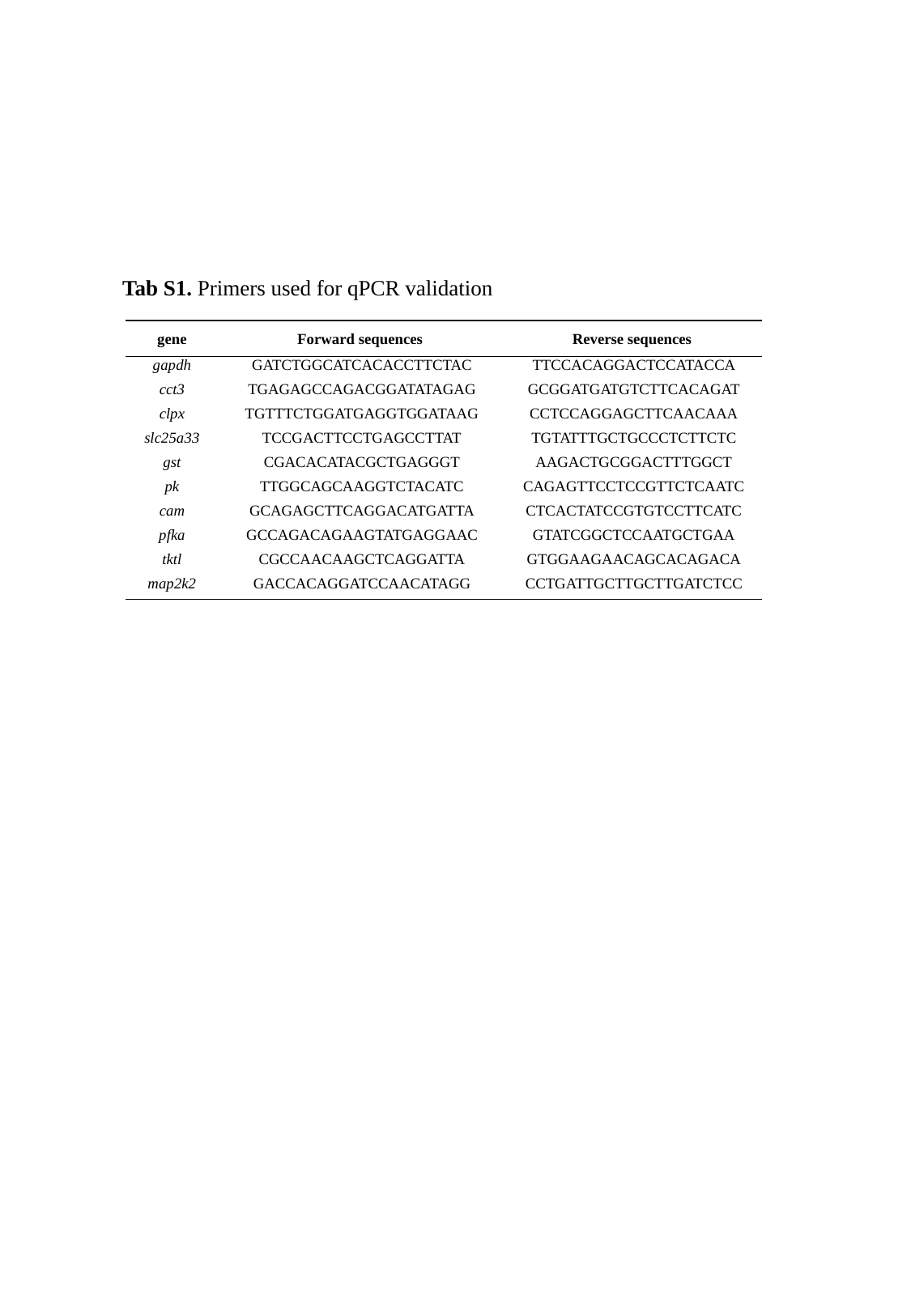

Tab S1. Primers used for qPCR validation
| gene | Forward sequences | Reverse sequences |
| --- | --- | --- |
| gapdh | GATCTGGCATCACACCTTCTAC | TTCCACAGGACTCCATACCA |
| cct3 | TGAGAGCCAGACGGATATAGAG | GCGGATGATGTCTTCACAGAT |
| clpx | TGTTTCTGGATGAGGTGGATAAG | CCTCCAGGAGCTTCAACAAA |
| slc25a33 | TCCGACTTCCTGAGCCTTAT | TGTATTTGCTGCCCTCTTCTC |
| gst | CGACACATACGCTGAGGGT | AAGACTGCGGACTTTGGCT |
| pk | TTGGCAGCAAGGTCTACATC | CAGAGTTCCTCCGTTCTCAATC |
| cam | GCAGAGCTTCAGGACATGATTA | CTCACTATCCGTGTCCTTCATC |
| pfka | GCCAGACAGAAGTATGAGGAAC | GTATCGGCTCCAATGCTGAA |
| tktl | CGCCAACAAGCTCAGGATTA | GTGGAAGAACAGCACAGACA |
| map2k2 | GACCACAGGATCCAACATAGG | CCTGATTGCTTGCTTGATCTCC |

## Slide 3
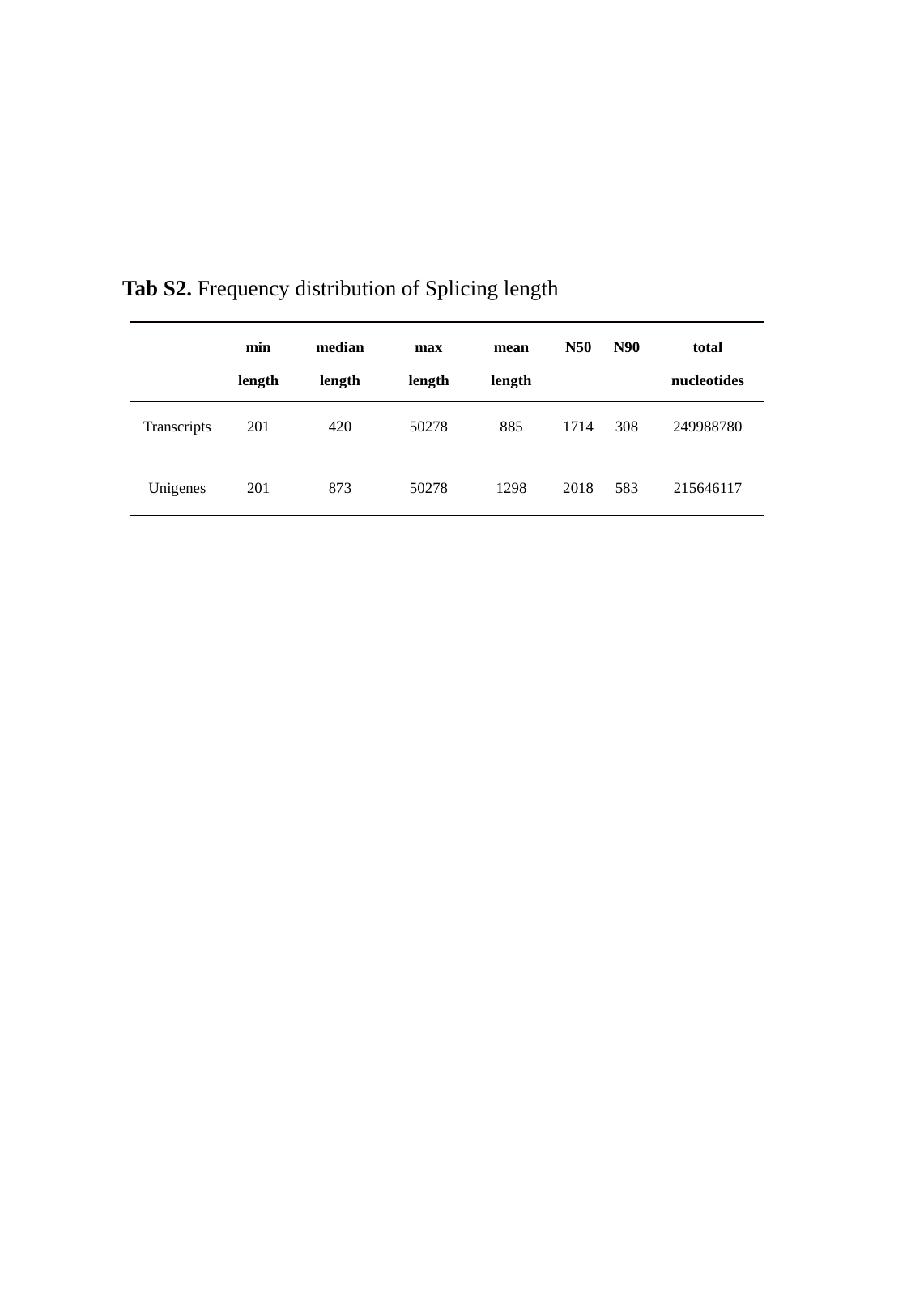

Tab S2. Frequency distribution of Splicing length
| | min length | median length | max length | mean length | N50 | N90 | total nucleotides |
| --- | --- | --- | --- | --- | --- | --- | --- |
| Transcripts | 201 | 420 | 50278 | 885 | 1714 | 308 | 249988780 |
| Unigenes | 201 | 873 | 50278 | 1298 | 2018 | 583 | 215646117 |

## Slide 4
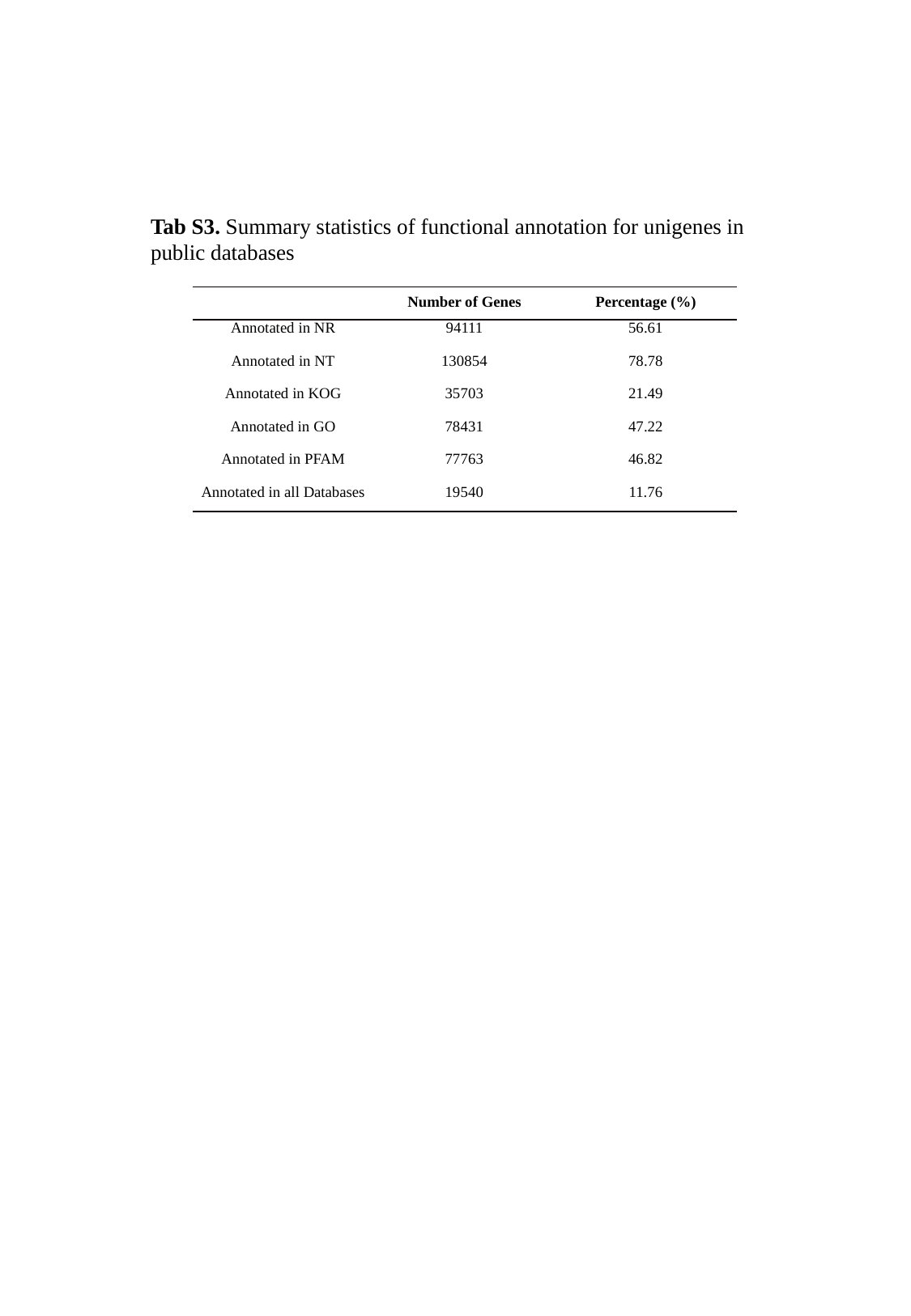

Tab S3. Summary statistics of functional annotation for unigenes in public databases
| | Number of Genes | Percentage (%) |
| --- | --- | --- |
| Annotated in NR | 94111 | 56.61 |
| Annotated in NT | 130854 | 78.78 |
| Annotated in KOG | 35703 | 21.49 |
| Annotated in GO | 78431 | 47.22 |
| Annotated in PFAM | 77763 | 46.82 |
| Annotated in all Databases | 19540 | 11.76 |
