# Supplementary material for: Morphological Characteristics and Comparative Transcriptome Analysis of Three Different Phenotypes of Pristella maxillaris
Source: Front Genet. 2019 Aug 2;10:698. doi: 10.3389/fgene.2019.00698 (PMC6687772; doi:10.3389/fgene.2019.00698)
Supplement: Supplementary file 2 [file Presentation_2.pptx]

## Slide 1
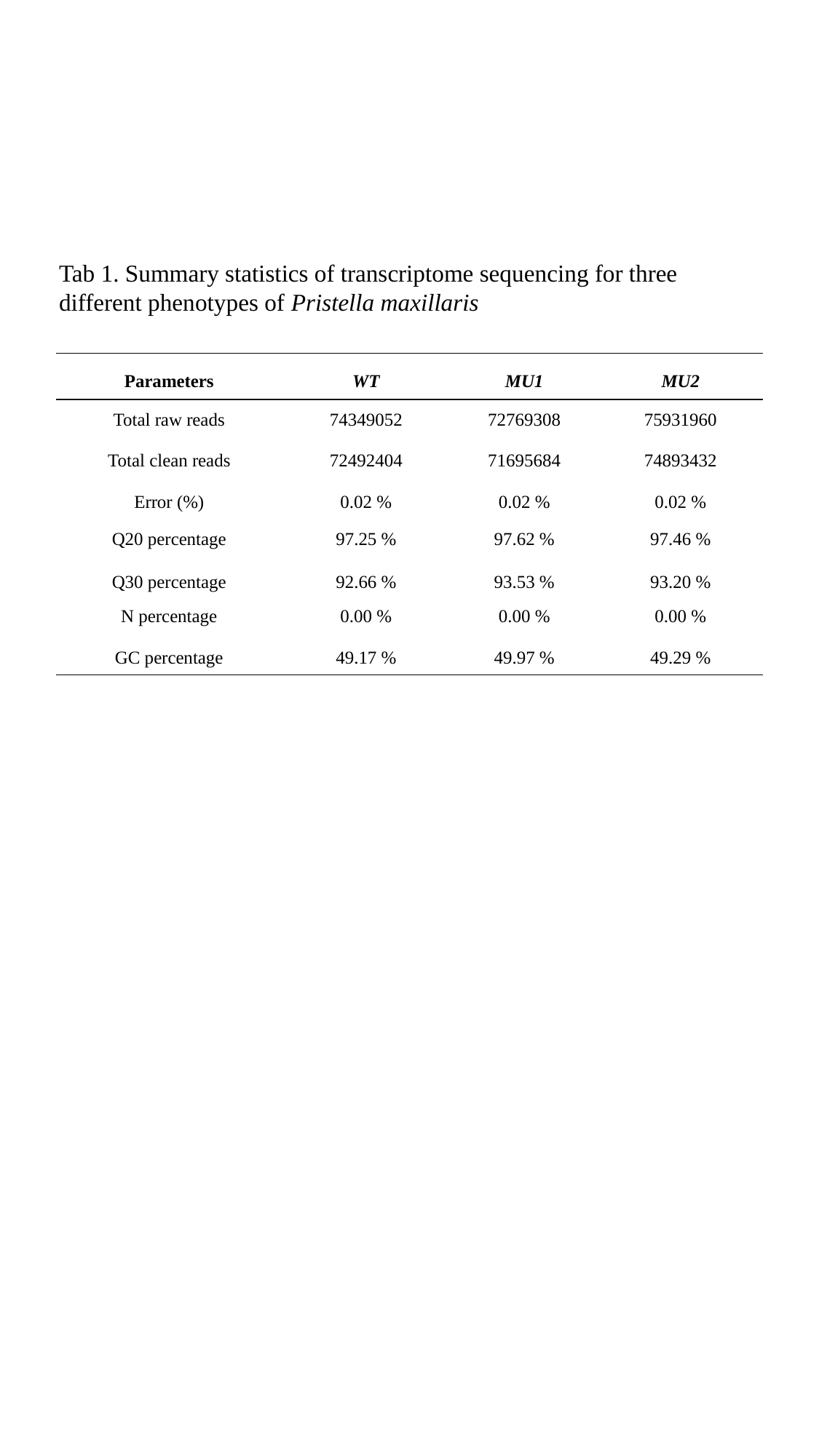

Tab 1. Summary statistics of transcriptome sequencing for three different phenotypes of Pristella maxillaris
| Parameters | WT | MU1 | MU2 |
| --- | --- | --- | --- |
| Total raw reads | 74349052 | 72769308 | 75931960 |
| Total clean reads | 72492404 | 71695684 | 74893432 |
| Error (%) | 0.02 % | 0.02 % | 0.02 % |
| Q20 percentage | 97.25 % | 97.62 % | 97.46 % |
| Q30 percentage | 92.66 % | 93.53 % | 93.20 % |
| N percentage | 0.00 % | 0.00 % | 0.00 % |
| GC percentage | 49.17 % | 49.97 % | 49.29 % |

## Slide 2
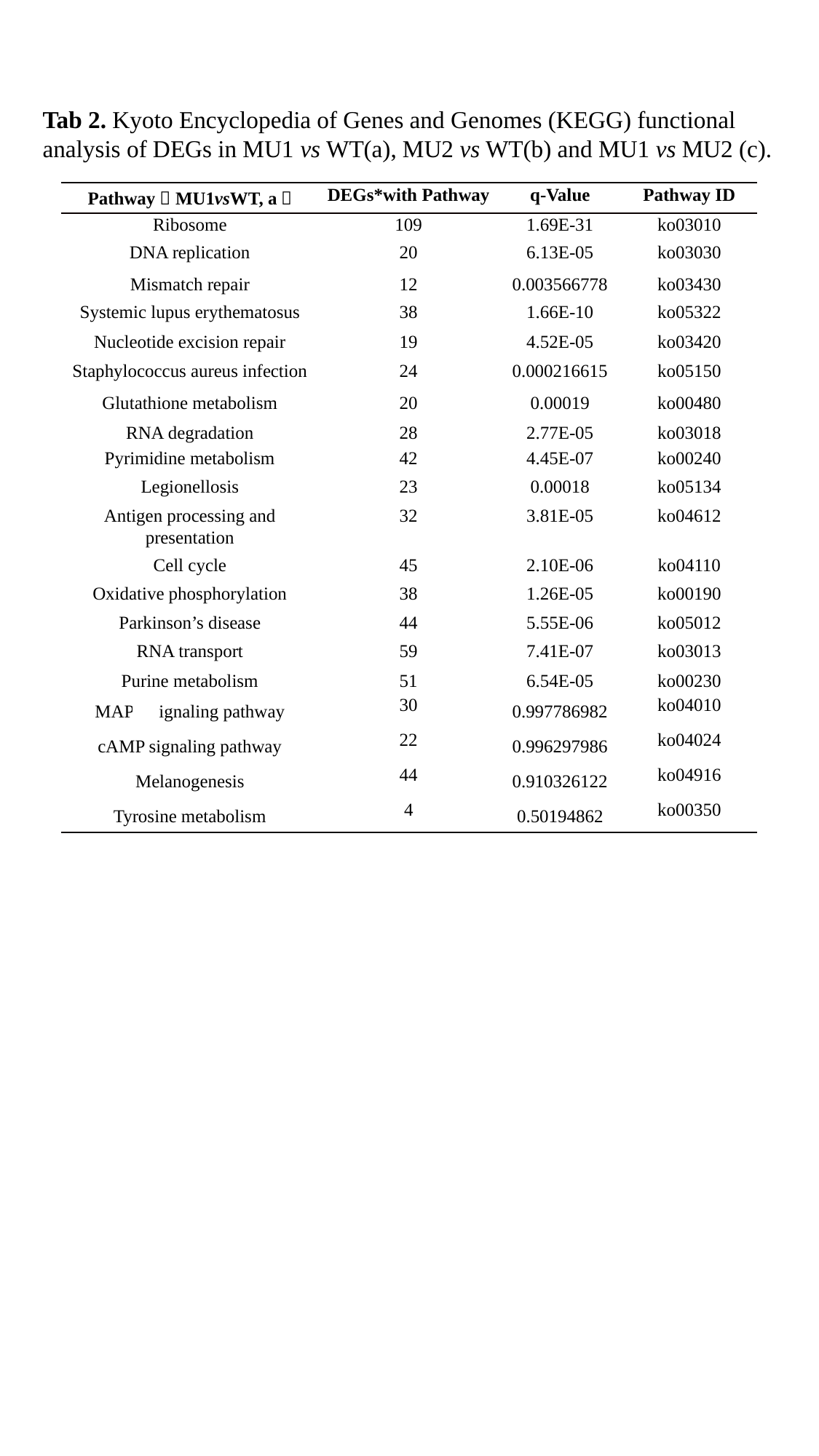

Tab 2. Kyoto Encyclopedia of Genes and Genomes (KEGG) functional analysis of DEGs in MU1 vs WT(a), MU2 vs WT(b) and MU1 vs MU2 (c).
| Pathway（MU1vsWT, a） | DEGs\*with Pathway | q-Value | Pathway ID |
| --- | --- | --- | --- |
| Ribosome | 109 | 1.69E-31 | ko03010 |
| DNA replication | 20 | 6.13E-05 | ko03030 |
| Mismatch repair | 12 | 0.003566778 | ko03430 |
| Systemic lupus erythematosus | 38 | 1.66E-10 | ko05322 |
| Nucleotide excision repair | 19 | 4.52E-05 | ko03420 |
| Staphylococcus aureus infection | 24 | 0.000216615 | ko05150 |
| Glutathione metabolism | 20 | 0.00019 | ko00480 |
| RNA degradation | 28 | 2.77E-05 | ko03018 |
| Pyrimidine metabolism | 42 | 4.45E-07 | ko00240 |
| Legionellosis | 23 | 0.00018 | ko05134 |
| Antigen processing and presentation | 32 | 3.81E-05 | ko04612 |
| Cell cycle | 45 | 2.10E-06 | ko04110 |
| Oxidative phosphorylation | 38 | 1.26E-05 | ko00190 |
| Parkinson’s disease | 44 | 5.55E-06 | ko05012 |
| RNA transport | 59 | 7.41E-07 | ko03013 |
| Purine metabolism | 51 | 6.54E-05 | ko00230 |
| MAPK signaling pathway | 30 | 0.997786982 | ko04010 |
| cAMP signaling pathway | 22 | 0.996297986 | ko04024 |
| Melanogenesis | 44 | 0.910326122 | ko04916 |
| Tyrosine metabolism | 4 | 0.50194862 | ko00350 |
| | |
| --- | --- |
| | |

## Slide 3
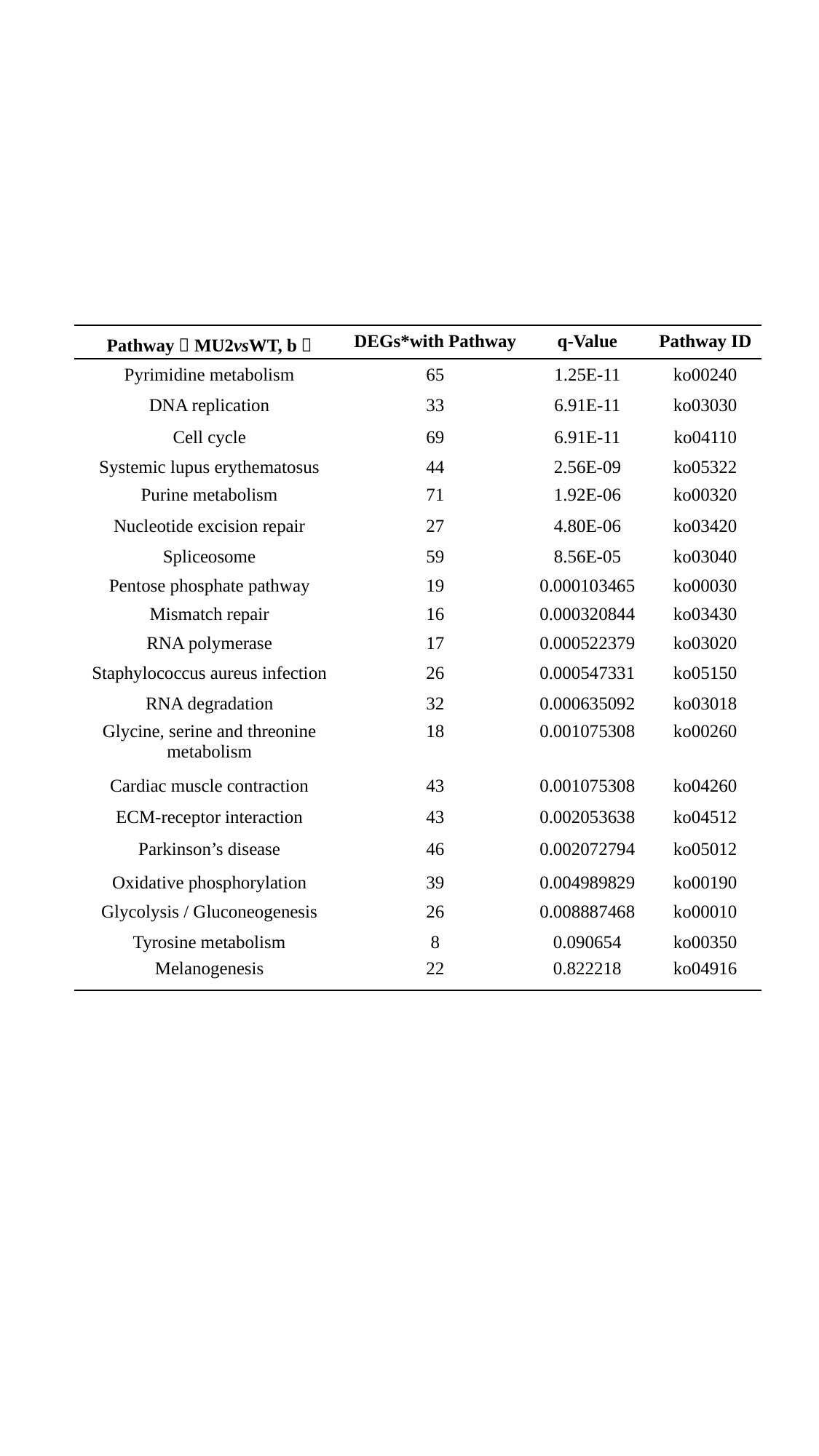

| Pathway（MU2vsWT, b） | DEGs\*with Pathway | q-Value | Pathway ID |
| --- | --- | --- | --- |
| Pyrimidine metabolism | 65 | 1.25E-11 | ko00240 |
| DNA replication | 33 | 6.91E-11 | ko03030 |
| Cell cycle | 69 | 6.91E-11 | ko04110 |
| Systemic lupus erythematosus | 44 | 2.56E-09 | ko05322 |
| Purine metabolism | 71 | 1.92E-06 | ko00320 |
| Nucleotide excision repair | 27 | 4.80E-06 | ko03420 |
| Spliceosome | 59 | 8.56E-05 | ko03040 |
| Pentose phosphate pathway | 19 | 0.000103465 | ko00030 |
| Mismatch repair | 16 | 0.000320844 | ko03430 |
| RNA polymerase | 17 | 0.000522379 | ko03020 |
| Staphylococcus aureus infection | 26 | 0.000547331 | ko05150 |
| RNA degradation | 32 | 0.000635092 | ko03018 |
| Glycine, serine and threonine metabolism | 18 | 0.001075308 | ko00260 |
| Cardiac muscle contraction | 43 | 0.001075308 | ko04260 |
| ECM-receptor interaction | 43 | 0.002053638 | ko04512 |
| Parkinson’s disease | 46 | 0.002072794 | ko05012 |
| Oxidative phosphorylation | 39 | 0.004989829 | ko00190 |
| Glycolysis / Gluconeogenesis | 26 | 0.008887468 | ko00010 |
| Tyrosine metabolism | 8 | 0.090654 | ko00350 |
| Melanogenesis | 22 | 0.822218 | ko04916 |

## Slide 4
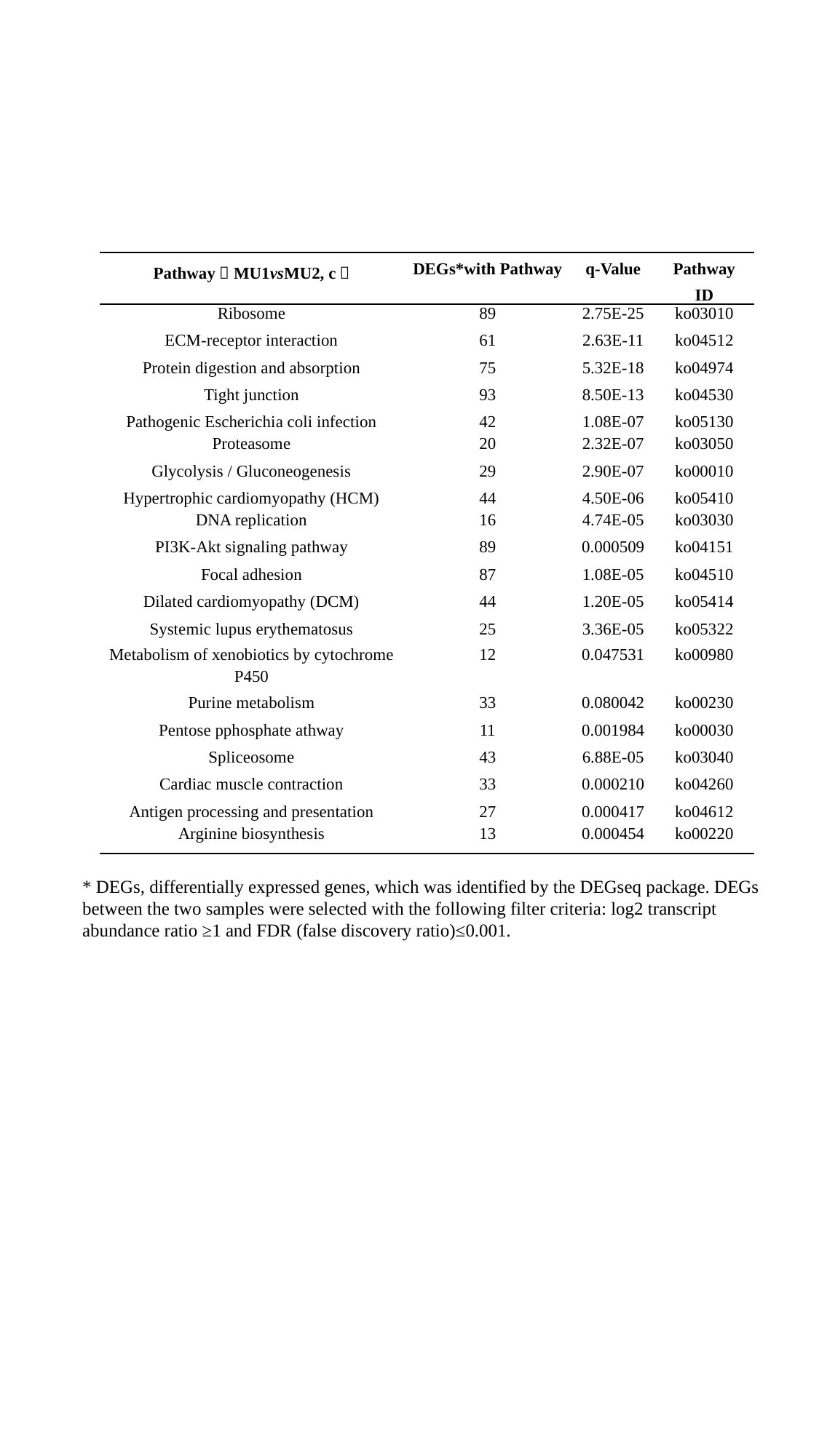

| Pathway（MU1vsMU2, c） | DEGs\*with Pathway | q-Value | Pathway ID |
| --- | --- | --- | --- |
| Ribosome | 89 | 2.75E-25 | ko03010 |
| ECM-receptor interaction | 61 | 2.63E-11 | ko04512 |
| Protein digestion and absorption | 75 | 5.32E-18 | ko04974 |
| Tight junction | 93 | 8.50E-13 | ko04530 |
| Pathogenic Escherichia coli infection | 42 | 1.08E-07 | ko05130 |
| Proteasome | 20 | 2.32E-07 | ko03050 |
| Glycolysis / Gluconeogenesis | 29 | 2.90E-07 | ko00010 |
| Hypertrophic cardiomyopathy (HCM) | 44 | 4.50E-06 | ko05410 |
| DNA replication | 16 | 4.74E-05 | ko03030 |
| PI3K-Akt signaling pathway | 89 | 0.000509 | ko04151 |
| Focal adhesion | 87 | 1.08E-05 | ko04510 |
| Dilated cardiomyopathy (DCM) | 44 | 1.20E-05 | ko05414 |
| Systemic lupus erythematosus | 25 | 3.36E-05 | ko05322 |
| Metabolism of xenobiotics by cytochrome P450 | 12 | 0.047531 | ko00980 |
| Purine metabolism | 33 | 0.080042 | ko00230 |
| Pentose pphosphate athway | 11 | 0.001984 | ko00030 |
| Spliceosome | 43 | 6.88E-05 | ko03040 |
| Cardiac muscle contraction | 33 | 0.000210 | ko04260 |
| Antigen processing and presentation | 27 | 0.000417 | ko04612 |
| Arginine biosynthesis | 13 | 0.000454 | ko00220 |
* DEGs, differentially expressed genes, which was identified by the DEGseq package. DEGs between the two samples were selected with the following filter criteria: log2 transcript abundance ratio ≥1 and FDR (false discovery ratio)≤0.001.

## Slide 5
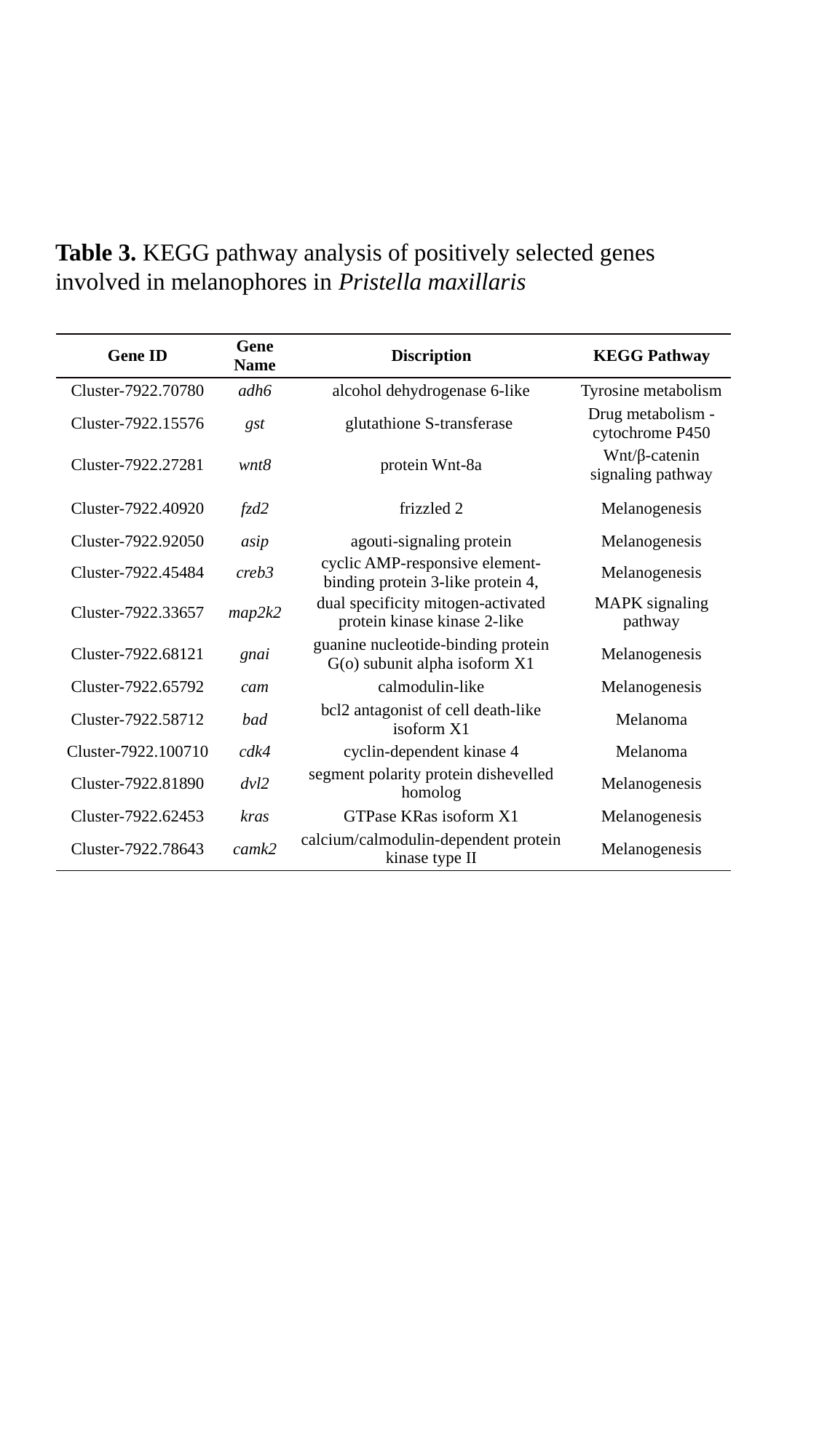

Table 3. KEGG pathway analysis of positively selected genes involved in melanophores in Pristella maxillaris
| Gene ID | Gene Name | Discription | KEGG Pathway |
| --- | --- | --- | --- |
| Cluster-7922.70780 | adh6 | alcohol dehydrogenase 6-like | Tyrosine metabolism |
| Cluster-7922.15576 | gst | glutathione S-transferase | Drug metabolism - cytochrome P450 |
| Cluster-7922.27281 | wnt8 | protein Wnt-8a | Wnt/β-catenin signaling pathway |
| Cluster-7922.40920 | fzd2 | frizzled 2 | Melanogenesis |
| Cluster-7922.92050 | asip | agouti-signaling protein | Melanogenesis |
| Cluster-7922.45484 | creb3 | cyclic AMP-responsive element-binding protein 3-like protein 4, | Melanogenesis |
| Cluster-7922.33657 | map2k2 | dual specificity mitogen-activated protein kinase kinase 2-like | MAPK signaling pathway |
| Cluster-7922.68121 | gnai | guanine nucleotide-binding protein G(o) subunit alpha isoform X1 | Melanogenesis |
| Cluster-7922.65792 | cam | calmodulin-like | Melanogenesis |
| Cluster-7922.58712 | bad | bcl2 antagonist of cell death-like isoform X1 | Melanoma |
| Cluster-7922.100710 | cdk4 | cyclin-dependent kinase 4 | Melanoma |
| Cluster-7922.81890 | dvl2 | segment polarity protein dishevelled homolog | Melanogenesis |
| Cluster-7922.62453 | kras | GTPase KRas isoform X1 | Melanogenesis |
| Cluster-7922.78643 | camk2 | calcium/calmodulin-dependent protein kinase type II | Melanogenesis |

## Slide 6
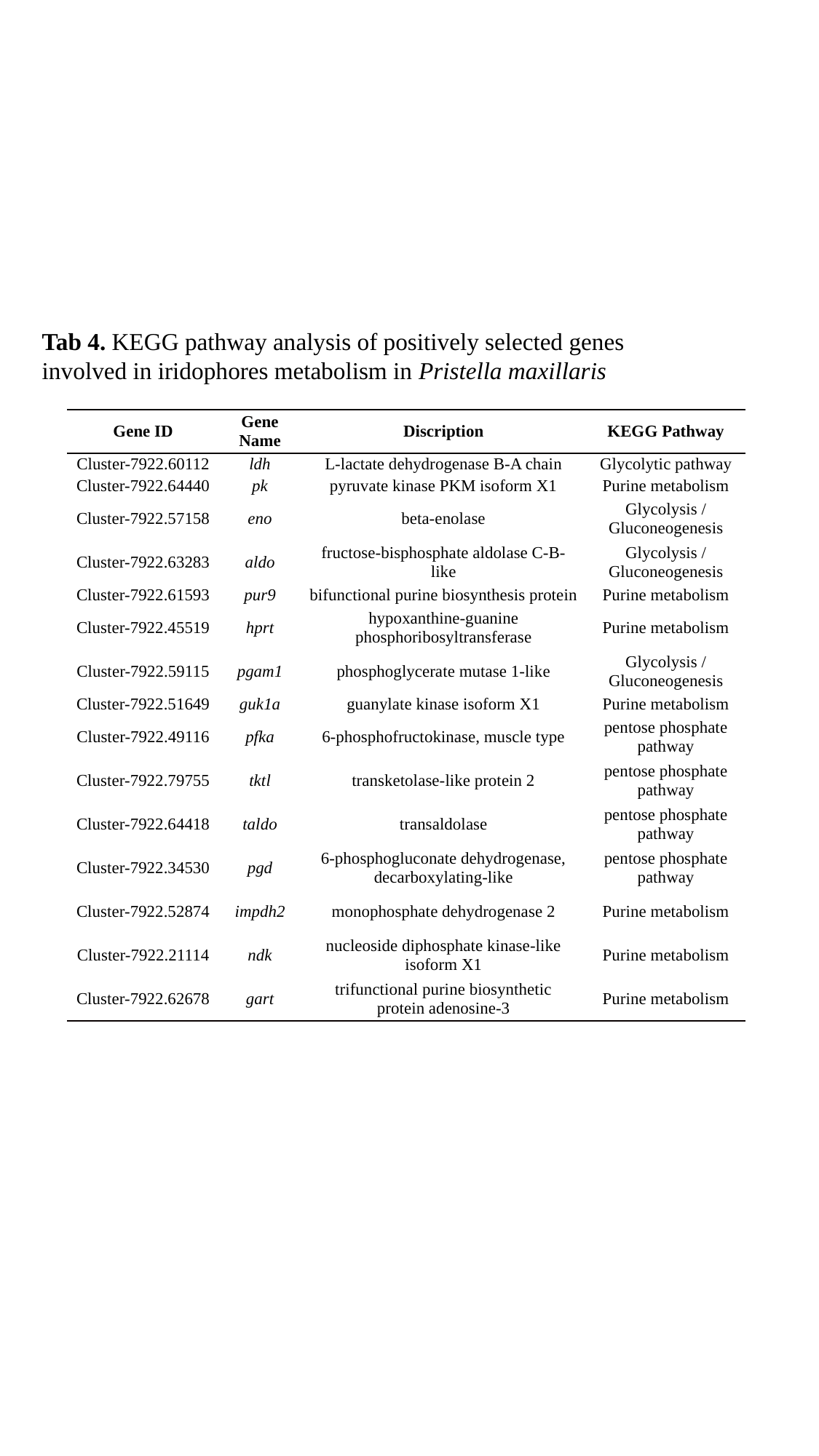

Tab 4. KEGG pathway analysis of positively selected genes involved in iridophores metabolism in Pristella maxillaris
| Gene ID | Gene Name | Discription | KEGG Pathway |
| --- | --- | --- | --- |
| Cluster-7922.60112 | ldh | L-lactate dehydrogenase B-A chain | Glycolytic pathway |
| Cluster-7922.64440 | pk | pyruvate kinase PKM isoform X1 | Purine metabolism |
| Cluster-7922.57158 | eno | beta-enolase | Glycolysis / Gluconeogenesis |
| Cluster-7922.63283 | aldo | fructose-bisphosphate aldolase C-B-like | Glycolysis / Gluconeogenesis |
| Cluster-7922.61593 | pur9 | bifunctional purine biosynthesis protein | Purine metabolism |
| Cluster-7922.45519 | hprt | hypoxanthine-guanine phosphoribosyltransferase | Purine metabolism |
| Cluster-7922.59115 | pgam1 | phosphoglycerate mutase 1-like | Glycolysis / Gluconeogenesis |
| Cluster-7922.51649 | guk1a | guanylate kinase isoform X1 | Purine metabolism |
| Cluster-7922.49116 | pfka | 6-phosphofructokinase, muscle type | pentose phosphate pathway |
| Cluster-7922.79755 | tktl | transketolase-like protein 2 | pentose phosphate pathway |
| Cluster-7922.64418 | taldo | transaldolase | pentose phosphate pathway |
| Cluster-7922.34530 | pgd | 6-phosphogluconate dehydrogenase, decarboxylating-like | pentose phosphate pathway |
| Cluster-7922.52874 | impdh2 | monophosphate dehydrogenase 2 | Purine metabolism |
| Cluster-7922.21114 | ndk | nucleoside diphosphate kinase-like isoform X1 | Purine metabolism |
| Cluster-7922.62678 | gart | trifunctional purine biosynthetic protein adenosine-3 | Purine metabolism |
